# Disrupted Frontoparietal Dynamics in Neurofibromatosis Type 1: Reduced Sensitivity and Atypical Modulation During Working Memory

**DOI:** 10.1002/hbm.70464

**Published:** 2026-02-03

**Authors:** Marta C. Litwińczuk, Shruti Garg, Caroline Lea‐Carnall, Nelson J. Trujillo‐Barreto

**Affiliations:** ^1^ School of Health Sciences University of Manchester Manchester UK; ^2^ Division of Psychology and Mental Health, Faculty of Biology, Medicine and Health University of Manchester Manchester UK; ^3^ Royal Manchester Children's Hospital Manchester University NHS Foundation Trust Manchester UK; ^4^ Geoffrey Jefferson Brain Research Centre Manchester Academic Health Science Centre Manchester UK; ^5^ School of Psychology Manchester Metropolitan University Manchester UK

**Keywords:** effective connectivity, functional MRI, Neurofibromatosis, working memory

## Abstract

Neurofibromatosis type 1 (NF1) is a rare, single‐gene neurodevelopmental disorder. Atypical brain activation patterns have been linked to working memory difficulties in individuals with NF1. This work investigates the alterations in frontoparietal effective connectivity in regions with atypical activation during working memory performance, with particular attention to self‐connections (intrinsic inhibitory influences each region exerts on itself). Forty‐three adolescents with NF1 and 26 age‐matched neurotypical controls completed functional magnetic resonance imaging scans during a verbal working memory task. Dynamic causal models (DCMs) were estimated for the bilateral frontoparietal network (dorsolateral and ventrolateral prefrontal cortices (dlPFC and vlPFC), superior and inferior parietal gyri (SPG and IPG)). The parametric empirical Bayes approach with Bayesian model reduction was used to test the hypothesis that NF1 diagnosis would be characterised by greater inhibitory intrinsic (self‐) connections. Leave‐one‐out cross‐validation (LOO‐CV) was performed to test the generalisability of group differences. NF1 participants demonstrated greater endogenous self‐connectivity of left dlPFC and IPG. The DCM that best explained the effects of working memory showed that the NF1 group has increased intrinsic connectivity of left vlPFC but weaker intrinsic connectivity of left dlPFC, left SPG and right IPG. The parameters of these connections showed a modest but positive predictive correlation coefficient of 0.34 (*p* = 0.002) with diagnosis status, suggesting a predictive value. Overall, increased endogenous self‐connectivity of left dlPFC and IPG in NF1 suggests reduced overall sensitivity of these regions to inputs. Working memory evoked different patterns of input processing in NF1 that cannot be characterised by increased inhibition alone. Instead, modulatory connectivity related to working memory showed less inhibitory self‐connectivity of left dlPFC, left SPG and right IPG and more inhibitory intrinsic connectivity of left vlPFC in NF1. This discrepancy between endogenous and modulatory connectivity suggests that overall NF1 participants are responsive to cognitive task‐related inputs but may show atypical adaptation to the task demands of working memory.

## Graphical abstract

Dynamic causal modelling of working memory fMRI in individuals with Neurofibromatosis Type 1 (NF1) revealed altered frontoparietal connectivity. NF1 participants showed increased endogenous self‐connectivity in left dlPFC and IPG. During working memory, NF1 participants showed increased self‐connectivity in left vlPFC but decreased self‐connectivity in left dlPFC, left SPG and right IPG.

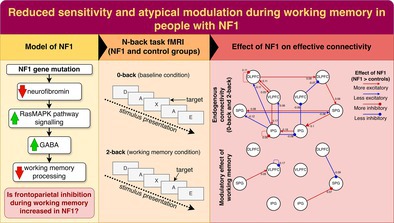

## 1 Introduction

Neurofibromatosis type 1 (NF1) is a rare, single‐gene, autosomal dominant neurodevelopmental disorder with a birth incidence of 1:2700 (Evans et al. 2010). Affected individuals present with a variety of symptom expression and severity, including pigmentary lesions (café‐au‐lait spots), dermal neurofibromas, skeletal abnormalities, brain and peripheral nerve tumours, learning disabilities and cognitive and social deficits (Gutmann et al. 2017). Individuals with NF1 also present with high rates of comorbidity with attention‐deficit/hyperactivity disorder and autism spectrum conditions (ASC) (Garg et al. 2012; Garg et al. 2013; Matson et al. 2013; van der Meer et al. 2012).

NF1 is caused by mutations to the NF1 gene, which encodes the neurofibromin protein. Mutations in the NF1 gene lead to reduced or aberrant neurofibromin function and consequently to hyperactivation of the RasMAPK signalling pathway (Daston and Ratner 2005; North 1997). This dysregulation has downstream effects of increasing presynaptic gamma‐aminobutyric acid (GABA) activity (Costa et al. 2002). Alterations in GABAergic activity are associated with disruption in neural development (van Lier et al. 2020). Additionally, GABA plays a crucial role in regulating neuronal activity through selective suppression of the firing of neurons (Bannai et al. 2015), and excessive GABA activity can disrupt the cortical excitation/inhibition balance (E/I) (Maffei et al. 2017). The E/I balance is essential for the maintenance function of the central nervous system and has further implications on the regional processing of cognitive tasks and the communication of input‐related signals between regions (Metkus et al. 2024; Sears and Hewett 2021; Sohal and Rubenstein 2019). Therefore, the selective effect of NF1 on GABAergic activity has broad implications for understanding the role of the E/I balance in the mechanisms of cognition and neurodevelopmental conditions.

NF1 presents with a complex profile of cognitive, behavioural and social disruptions. Research comparing NF1 individuals to their unaffected siblings documented academic underperformance and cognitive difficulties (Lehtonen et al. 2012), including the domains of general intelligence, visuospatial processing, executive function, attention and social cognition. Empirical research has reliably demonstrated working memory difficulties in NF1 (Lehtonen et al. 2012; Pobric et al. 2021; Sawyer et al. 2022; Shilyansky et al. 2010), which is the ability to temporarily maintain and manipulate information in one's memory (Baddeley 1992; Baddeley et al. 2014). Working memory underlies our ability to perform complex tasks and it is critical for academic achievement, including language acquisition, reading comprehension and mathematics performance (Gathercole and Alloway 2005). Consequently, the neural and cognitive mechanisms of working memory disruptions in NF1 may contribute to their broader academic and cognitive challenges.

Several neuroimaging studies have highlighted the effects of NF1 on neural function during working memory. In a seminal study, Shilyansky et al. (2010) conducted parallel neuroimaging research of working memory performance and learning in humans and mice with the NF1 mutation. Human research showed that compared to neurotypical controls, NF1 participants were less accurate and slower during performance of a working memory task. The authors also investigated the neural correlates of these disruptions in NF1 with functional magnetic resonance imaging (fMRI). Compared to neurotypical controls, the NF1 group had weaker activation in parts of the distributed working memory network—including the dorsolateral prefrontal cortex (dlPFC), frontal eye fields, parietal cortex and striatum. These findings are paralleled by electrophysiological findings of elevated inhibitory activity in prefrontal cortical and striatal regions from Nf1+/− mouse models. Therefore, Shilyansky et al. (2010) proposed that these patterns of regional hypoactivation in human frontal and parietal regions may be directly linked to increased GABAergic inhibition. Ibrahim et al. (2017) extended these findings with the investigation of communication within the frontoparietal network. By investigating functional connectivity during working memory performance with the generalized psychophysiological interaction (gPPI) method, Ibrahim et al. (2017) demonstrated that in response to increased working memory load, the neurotypical control group had greater connectivity between the right parietal seed and critical working memory‐related regions, including bilateral parietal cortex, and frontal areas (right pars opercularis and left premotor cortex). This suggests that individuals with NF1 may have difficulty in modulating their connectivity within the frontoparietal network in response to increasing working memory demands.

The next step to understanding the mechanisms of working memory performance in individuals with NF1 is to investigate how E/I balance relates to the patterns of hypoactivity during working memory tasks. To achieve this, we identified patterns of hypoactivity in the frontoparietal network in fMRI data collected during a verbal working memory task (N‐back task) from adolescents diagnosed with NF1 and age‐matched neurotypical controls. Then, we applied dynamic causal modelling (DCM) to these regions. DCM is a framework that allows the estimation of directed interactions (both excitatory and inhibitory) between neuronal populations, known as effective connectivity (Zeidman, Jafarian, Corbin, et al. 2019). In addition, and of particular interest to this work, DCM also estimates intrinsic connectivity, which reflects the self‐inhibitory dynamics of a region and, by extension, its responsiveness (sensitivity) to external inputs (Snyder et al. 2021). Importantly, intrinsic (self‐) connectivity serves as a proxy for the region's E/I balance, which is regulated by GABAergic neurotransmission (Maffei et al. 2017). In the context of NF1, elevated GABA levels have been proposed to disrupt this balance (Buratti et al. 2012; Payne et al. 2010; Petroff 2016). This may lead to increased inhibitory signalling within the region and its hypoactivity during tasks. Based on this evidence, we hypothesize that the NF1 group will have increased intrinsic (self‐) connectivity in the frontoparietal network during N‐back tasks compared to neurotypical controls.

## 2 Methods

### 2.1 NF1 Participants

The adolescents aged 11–17 years were recruited via the Northern UK NF—National Institute of Health, with (i) diagnostic criteria [National Institutes of Health Consensus Development Conference. Neurofibromatosis conference statement. Arch. Neurol. 45, 575–578 (1988)] and/or molecular diagnosis of NF1; (ii) no history of intracranial pathology other than asymptomatic optic pathway or other asymptomatic and untreated NF1‐associated white matter lesion or glioma; (iii) no history of epilepsy or any major mental illness and (iv) no MRI contraindications. Participants on pre‐existing medications such as stimulants, melatonin or selective serotonin reuptake inhibitors were not excluded from participation. The study was conducted in accordance with local ethics committee approval (Ethics reference: 18/NW/0762, ClinicalTrials.gov Identifier: NCT0499142. Registered 5th August 2021; retrospectively registered, https://clinicaltrials.gov/ct2/show/NCT04991428). All methods were carried out in accordance with relevant guidelines and regulations.

Thirty‐three participants with NF1 were recruited in this way and included in a previous study described in detail in Garg et al. (2022), and 19 new participants with NF1 were added to this dataset. Seven NF1 participants withdrew from the study, and two participants had data with high motion/scanning artifacts. The final sample consisted of 43 individuals with NF1. Table 1 details the characteristics of the remaining NF1 sample, including the presence of tumours, medication, child adaptive behaviour and pre‐existing diagnoses.

**TABLE 1:** Characteristics of the 43 participants with NF1 diagnosis. Child adaptive behaviour was assessed with parent‐rated Vineland Adaptive Behaviour Scale—third edition (Hill et al. 2017).

Tumours (*n*)	8
Medication (*n*)	10
Vineland adaptive behaviour composite (mean, standard deviation)	73.19 (19.25)
Pre‐existing diagnoses (*n*)	
No comorbidity	21
ADHD	5
ADHD, Anxiety	1
ADHD, ASD, Anxiety	1
ADHD, LD	1
Anxiety	1
ASD	4
ASD, ADHD	1
ASD, ADHD, LD	1
Auditory Processing Disorder	1
LD	6

Abbreviations: ADHD, attention deficit hyperactivity disorder; ASD, autism spectrum disorder; LD, learning difficulty.

### 2.2 Neurotypical Controls

Twenty‐nine adolescents with no diagnosis of NF1 were recruited. Two neurotypical control participants did not complete their fMRI scanning; one participant's data were corrupted by an artefact. Controls' age and sex were matched to the sample of the NF1 group. Table 2 includes demographic information for NF1 and control groups.

**TABLE 2:** Demographic data of study participants (43 NF1 participants, 26 neurotypical controls). SD = standard deviation; M = male; F = Female.

	Mean age (SD)	Sex (M/F)
NF1	14.8 (1.83)	22/21
Controls	14.15 (2.71)	12/14

### 2.3 Experimental Procedure

Participants lay in the scanner and a T1‐weighted image was acquired. Participants were asked to perform a working memory task for 6 min while fMRI scans were acquired. In addition, T2‐weighted images were acquired at the first visit (after the T1 image) and reviewed by a paediatric neuro‐radiologist to rule out NF1‐associated tumours.

**FIGURE 1:**
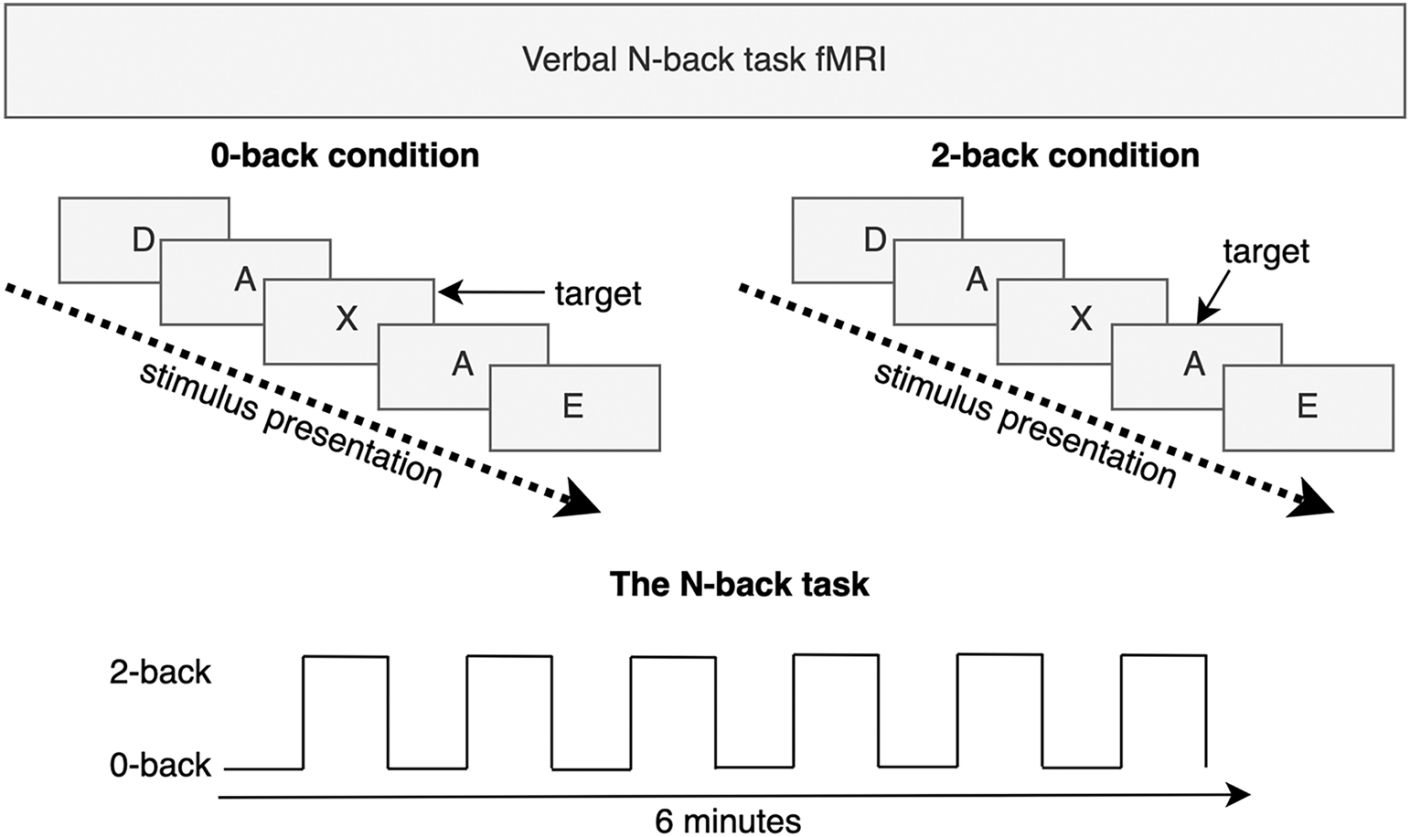
A visual illustration of the verbal working memory task that the participants performed during scanning.

The working memory task session consisted of six blocks, each of 0‐back and 2‐back verbal N‐back task (Figure 1). Each block was 30 s long and consisted of nine trials. Trials repeated every 3 s; stimuli were presented for 500 milliseconds (ms), with an interstimulus interval of 2500 ms. During the 0‐back condition, participants were instructed to press a hand‐held button only when the letter ‘X’ was presented on the screen. For the 2‐back condition, the participants were instructed to press the same button when the letter on the screen matched the letter 2 presentations before. Before each block, an instruction screen displayed either ‘0‐back’ or ‘2‐back’ for 2000 ms to inform the participants about the upcoming condition block. Before entering the scanner, the participants received task instructions from the researcher and completed a practice session that was monitored to ensure they understood the task requirements.

### 2.4 Image Acquisition

Imaging was performed on a 3‐Tesla Philips Achieva scanner using a 32‐channel head coil with a SENSE factor of 2.5. To maximise signal‐to‐noise (SNR), we utilised a dual‐echo fMRI protocol developed by Halai et al. (2014). The fMRI sequence included 36 slices, 64 × 64 matrix, field of view (FOV) 224 × 126 × 224 mm, in‐plane resolution 2.5 × 2.5 mm, slice thickness 3.5 mm, TR = 2.5 s, TE = 12 ms and 35 ms. The total number of volumes collected for each fMRI run was 144.

### 2.5 Image Processing

Image processing was done using SPM12 (Wellcome Department of Imaging Neuroscience, London; http://www.fil.ion.ucl.ac.uk/spm) and MATLAB R2023a. Dual echo images were extracted and averaged using in‐house MATLAB code developed by Halai et al. (2014) (DEToolbox). First, functional images were slice‐time corrected and realigned to the first image. Then, the short and long echo times were combined for each timepoint. The orientation and location of the origin point of every anatomical T1 image were checked and corrected where needed. The mean functional EPI image was co‐registered to the structural (T1) image. Motion parameters estimated during co‐registration of short images were used as input to the Artifact Detection Tools (ART; https://www.nitrc.org/projects/artifact_detect/) toolbox, along with combined dual echo scans for identification of outlier and motion‐corrupted images across the complete scan. The outlier detection threshold was set to changes in global signal 3 standard deviations away from the mean global brain activation. The motion threshold for identifying scans to be censored was set to 3 mm. Outlier images and images corrupted by motion were censored during the analysis by using the outlier volume regressors. Participants who had 29 or more volumes censored (≥ 20% of total fMRI time series) due to excessive motion or signal outliers were removed from the analysis. Following this quality control, no controls were removed from the analysis and two NF1 participants were removed (26 controls and 43 NF1 participants remained). Unified segmentation was conducted to identify grey matter, white matter and cerebrospinal fluid. Normalisation to MNI space was done with diffeomorphic anatomical registration using exponentiated Lie algebra (DARTEL) (Ashburner 2007) registration method for fMRI. Normalised images were interpolated to isotropic 2 × 2 × 2 mm voxel resolution. A 6 × 6 × 6 mm full width at half maximum (FWHM) Gaussian smoothing kernel was applied.

### 2.6 Behavioural Analysis

Behavioural performance during the N‐back task was described by accuracy, response times (RT), inverse efficiency score (IES) (Bruyer and Brysbaert 2011) and D‐prime (Macmillan and Creelman 2004). Accuracy was calculated as (correct hits + correct omissions)/total trials. RT was calculated for correct responses to target stimuli. The inverse efficiency score (IES) was calculated by dividing RT by accuracy as a measure of speed‐accuracy trade‐off, in which lower scores reflect better cognitive performance (faster response at lower accuracy cost) (Bruyer and Brysbaert 2011). D‐prime was calculated as a measure of sensitivity, representing the ability to discriminate between target and nontarget stimuli while accounting for response bias. It was calculated as z(hit rate)−z(false alarm rate), where z represents the inverse of the standard normal cumulative distribution function (Macmillan and Creelman 2004). The verbal N‐back data for seven control participants were unavailable.

Group differences between control and NF1 groups were analysed using general linear models (GLMs); t‐statistic was obtained for group effect, while controlling for age and sex as covariates. Separate GLM models were constructed for each behavioural measure in each condition. Statistical significance threshold alpha was set to 0.05.

### 2.7 fMRI Analysis

**FIGURE 2:**
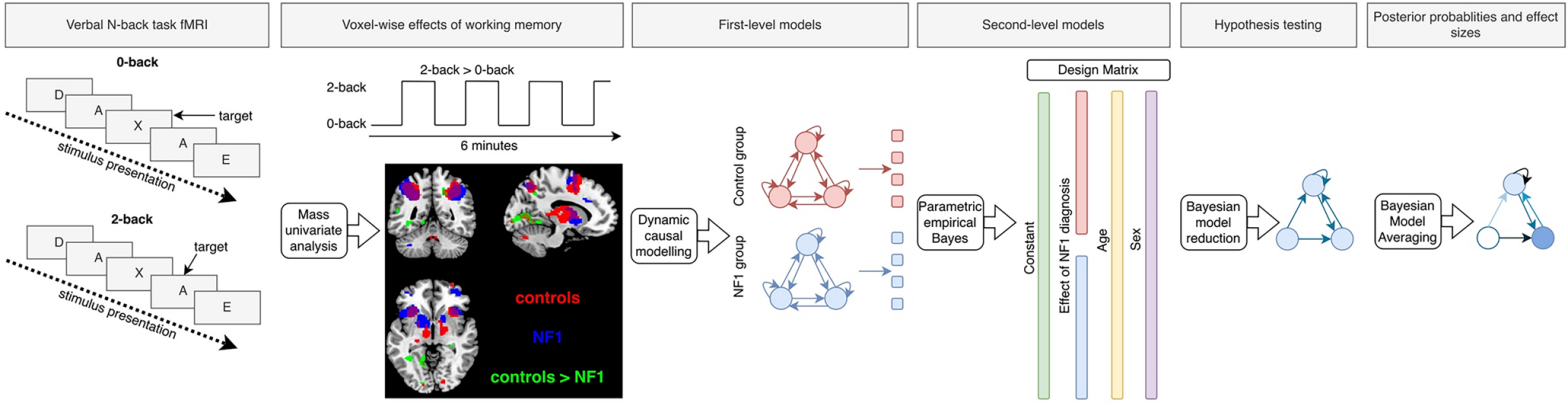
A visual summary of the fMRI analysis pipeline. In summary, the mass univariate analysis was performed to identify the effect of working memory in control and NF1 groups (2‐back > 0‐back contrast), and then the areas where working memory evokes greater activity in controls than NF1 were identified (controls > NF1 contrast). Volumes showing these group effects were carried to the first‐level DCM. Group effects on effective connectivity were identified with the parametric empirical Bayes approach. Connections that had no contribution to the model fit were removed with Bayesian model reduction. Finally, connection weights were averaged with Bayesian model averaging.

The fMRI analysis and connectivity modelling pipeline has been visually summarised in Figure 2.

For each subject, a canonical hemodynamic response function was fitted for 0‐back and 2‐back conditions of the N‐back task, and a general linear model (GLM) was estimated. A high‐pass filter with a cut‐off at 128 s was applied to remove slow signal drifts. An autoregressive model (AR(1)) was fitted to estimate and remove serial correlations. The global masking threshold remained at the default value of 0.8. The volume censoring regressors and six motion parameters were included as covariates in the model. One F‐contrast of the main effect of condition was defined, followed by a 2‐back > 0‐back t‐contrast.

First, we investigated the shared effect of working memory in the whole sample of both NF1 and control groups together (2‐back > 0‐back contrast). Next, we estimated the effect of working memory separately in each group and created a union mask (uncorrected *p* value = 0.001). Within this union mask, we compared the effect of working memory across NF1 and control groups with whole‐brain mass univariate analysis (controls > NF1 group contrast). Age and sex were included in the design matrix as covariates of no interest.

### 2.8 Dynamic Causal Modelling (DCM)

DCM is a Bayesian framework developed to infer and quantify effective connectivity, the directed causal influence that one brain region exerts over another during experimental tasks. It can provide a mechanistic model of how observed BOLD signals arise from underlying neuronal interactions. Here, DCM for fMRI was used to estimate effective connectivity between regions during the N‐back task (Friston et al. 2003). It uses a generative model of neuronal and hemodynamic state equations to predict the BOLD signal. This allows inference of the underlying neural activity (Zeidman, Jafarian, Seghier, et al. 2019). In DCM, the A‐matrix describes the baseline or endogenous connectivity during the whole task, the B‐matrix describes the task‐dependent modulations of these connections, and the C‐matrix describes how external inputs drive activity within specified regions. The off‐diagonal elements of the A and B matrices represent extrinsic connectivity (between‐region influences). The diagonal elements of the A and B matrices represent the intrinsic connections or self‐connections (the inhibitory influence each region exerts on itself). The B‐matrix captures task‐dependent modulations of these connections, while the C‐matrix represents direct experimental inputs.

Importantly, the main manuscript focuses on modelling of brain regions that show reduced effect of working memory in the frontoparietal network in the NF1 group (i.e., hypoactivity), as compared to the control group. The Supporting Information 1 presents the atypical connectivity within the regions commonly activated in controls and NF1 groups (Seghier et al. 2010). The examination of the A‐matrix reveals alterations in NF1 that affect the general network organisation that persists across the two cognitive states measured here (0‐back and 2‐back). The examination of the B‐matrix will reveal condition‐specific differences that emerge due to the modulatory effects of working memory. The B‐matrix needs to be interpreted relative to the A‐matrix to understand whether NF1 affects the baseline network architecture, or whether it alters the magnitude of condition‐dependent responses in otherwise preserved network architecture.

### 2.9 Volumes of Interest

Eight volumes of interest (VOIs) were defined in the frontal and parietal lobes. VOIs included bilateral superior parietal gyrus (SPG), bilateral ventrolateral prefrontal cortex (vlPFC) and bilateral dorsolateral prefrontal cortex (dlPFC). Their centre coordinates were placed based on the peak group differences in the effect of working memory (controls > NF1 group contrast). For the bilateral inferior parietal gyrus (IPG), this region was added based on peak activation during the task across all participants because it would serve as the region that receives the driving input based on prior research (Ma et al. 2011). VOIs are consistent with meta‐analyses of verbal and identity monitoring working memory (Emch et al. 2019; Owen et al. 2005; Yaple et al. 2019).

Table 3 lists the MNI coordinates where VOI spheres were placed. VOIs were defined as spheres with a radius of 6 mm, and they were additionally masked by the union mask of the effect of working memory, derived from the mass univariate analysis. This ensured that models reflect shared and unique patterns of neuronal activity related to working memory, rather than background activity.

**TABLE 3:** The MNI coordinates of the centre of the VOI spheres.

	Left	Right
SPG	[−20, −60, 40]	[20, −56, 48]
vlPFC	[−32, 24, 8]	[34, 24, 4]
dlPFC	[−48, 32, 22]	[47, 32, 24]
IPG	[−32, −44, 38]	[32, −42, 24]

For every participant, the first eigenvariate of the timeseries of all voxels in each VOI was extracted and adjusted by F‐contrast of main effects of the N‐back task, which removed effects of motion and outlier scans from the timeseries.

### 2.10 First‐Level Analysis

We specified a deterministic and bilinear DCM in which neural dynamics are driven by the task inputs and the experimental conditions modulate the strength of the connections. The driving input (C‐matrix) represents the main effect of the task, meaning the onset of all trials. Based on the prior literature, the driving input entered the bilateral IPG (Ma et al. 2011). Additionally, the modulatory input was the effect of working memory load on all connections. Each model was fully connected within the hemisphere, and each region was connected to its contralateral homologue—this resulted in 40 intrinsic and modulatory connections between the eight VOIs.

### 2.11 Second‐Level Analysis

Our primary hypothesis focused on differences in self‐connections, reflecting self‐inhibitory dynamics of a region, during working memory in NF1. Following the inversion of first‐level DCMs, we performed second‐level analysis using the parametric empirical Bayes (PEB) approach (Friston et al. 2016; Zeidman, Jafarian, Seghier, et al. 2019). This revealed the group mean connectivity shared across the control and NF1 groups, and the effect of NF1 diagnosis on connectivity. The PEB analysis was performed separately for the A‐matrix and B‐matrix. We included age and sex as covariates of no interest, and we mean‐centred the design matrix.

Next, we investigated our hypothesis that the NF1 diagnosis would be associated with increased strength of inhibitory self‐connections during working memory in the NF1 group (intrinsic connectivity in the B‐matrix). Therefore, we sequentially ‘switched off’ self‐connections of every region and compared model evidence between the full and reduced (nested) models, which differed only in their priors. We used Bayesian Model Reduction (BMR) to estimate the free energy (an approximation of a model's log‐evidence) of reduced models and their corresponding parameters. This enabled Bayesian model comparison (BMC) by comparing the free energy of each model (Friston et al. 2016). Next, Bayesian model averaging (BMA) was used to obtain the average of parameter estimates across models weighted by each model's posterior probability (Penny et al. 2007). In DCM, the posterior probability can refer either to the overall model evidence (via free energy) or to individual parameters (i.e., the probability that a connection differs from zero). Posterior probability parameters of individual connections represent the effect sizes (Friston et al. 2016). The evidence level of the models is interpreted according to Kass and Raftery (1995), where posterior probability > 0.5 is considered weak evidence, > 0.75 is considered positive evidence and > 0.95 is considered strong evidence.

Finally, leave‐one‐out cross‐validation (LOO‐CV) was used to assess how well the estimated brain connectivity patterns could predict diagnosis status. At each fold, the PEB approach was repeated using all participants except one to estimate group‐level connectivity parameters for intrinsic (self‐) connections identified via BMC. The diagnosis for the held‐out participant was then predicted based on these estimated parameters. This process was repeated until each participant had been excluded once.

## 3 Results

### 3.1 Behaviour

Table 4 summarises the descriptive statistics of each group's performance. During the 0‐back condition, there were no significant group differences for accuracy (*t*(58) = −0.34, *p* = 0.733), response time (*t*(58) = 1.63, *p* = 0.108), inverse efficiency score (*t*(58) = 1.42, *p* = 0.162) or d‐prime (*t*(58) = −0.16, *p* = 0.877). During the 2‐back condition, individuals with NF1 showed significantly reduced accuracy (*t*(58) = −2.05, *p* = 0.044), and d‐prime (*t*(58) = −3.21, *p* = 0.002). The inverse efficiency score showed a trend towards significantly poorer speed‐efficiency trade‐off in the NF1 group (*t*(58) = 1.94, *p* = 0.058). Response times showed no significant group differences (*t*(58) = 1.42, *p* = 0.162).

**TABLE 4:** A summary of control and NF1 groups' performance during N‐back task. M = mean; SD = standard deviation; ms = milliseconds.

Measure	Condition	Control group		NF1 group	
		M	SD	M	SD
Accuracy	0‐back	0.98	0.07	0.97	0.12
	2‐back	0.96	0.04	0.92	0.09
Response time (ms)	0‐back	553.9	111.3	617	188
	2‐back	674	169.7	744.7	224.5
Inverse efficiency score	0‐back	572.3	138.1	667.4	304.7
	2‐back	701.4	184	818.1	259.4
D‐prime	0‐back	3.79	0.84	3.74	0.9
	2‐back	3.82	0.44	3.17	0.82

### 3.2 Mass Univariate Analysis

Details of all local activation peaks, described in this section, are provided in Supporting Information 2.

The effects of working memory (2‐back > 0‐back contrast) were evaluated separately for NF1 and control groups. In both groups, working memory engagement was associated with bilateral activation in frontal regions, including superior, middle and inferior frontal gyri. Additional activation was observed in the precentral gyri and supplementary motor area. In the parietal cortex, both groups showed activation in IPG and precuneus. Other regions showing significant working memory effects included the left insula and the left globus pallidus.

**FIGURE 3:**
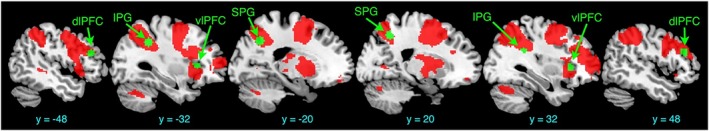
A binarized overlay of clusters generated by mass univariate analysis showing regions where the control group has greater working memory‐related activation than the NF1 group. Green voxels illustrate the VOIs. The cyan y‐coordinates are the MNI coordinates of the sagittal slices.

Next, group differences were examined with the controls > NF1 contrast. Figure 3 illustrates the binarized mask of controls > NF1 contrast and the location of the VOIs. As hypothesized, working memory evoked greater activation in controls in the frontoparietal network, including bilateral SPG, bilateral vlPFC and left dlPFC (all passing the uncorrected *p* value threshold of 0.001). There was a relatively weak but significant difference in activation of the left IPG, left supramarginal gyrus and right angular gyrus. Differences in activation also extended over bilateral insula, left middle cingulate cortex, left calcarine fissure, left middle temporal gyrus and right cerebellum.

### 3.3 Effective Connectivity

The PEB approach was separately used to estimate endogenous connectivity (A‐matrix) and modulatory connectivity (B‐matrix). The following sections describe group differences that have strong evidence (posterior probability of including the connection in the model > 0.95%), while Supporting Information 3 presents tables with all connection values and their posterior probabilities.

#### 3.3.1 Endogenous Connectivity

**FIGURE 4:**
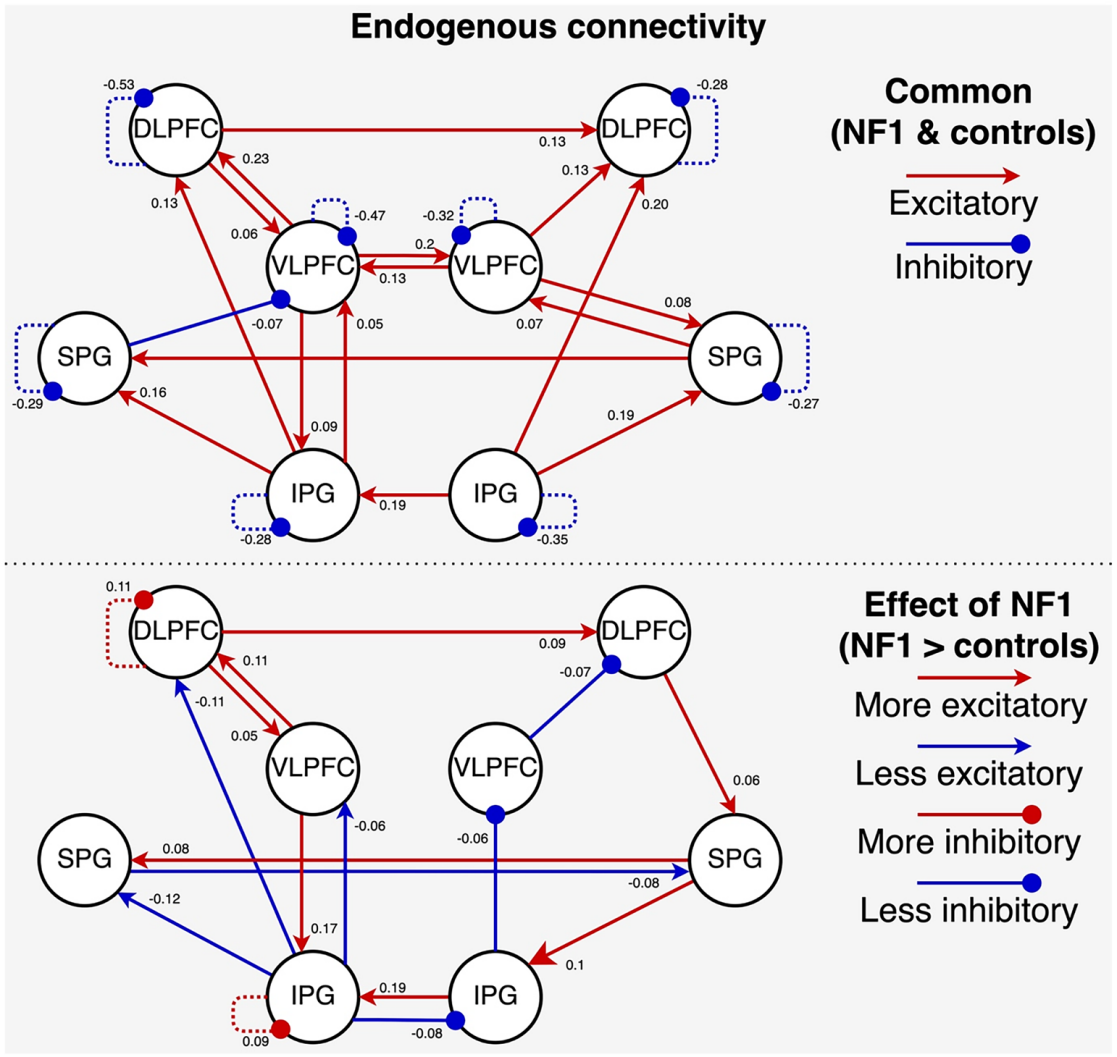
The endogenous connectivity during 0‐back and 2‐back conditions of the N‐back task. The top panel shows connections common to both groups while the bottom panel shows group differences (NF1 > controls). All connections illustrated here exceed the 95% posterior probability threshold and have strong evidence of being included in the model. In the top graph, red connections ending in an arrowhead indicate excitatory connections, and blue connections ending with a dot indicate inhibitory connections. In the bottom panel, red colour indicates that connections are stronger in NF1, and blue indicates that connections are weaker in NF1. For intrinsic (self‐) connections, ‘stronger’ indicates greater self‐inhibition.

The top graph in Figure 4 illustrates common (i.e., shared) connection strength, while the bottom graph illustrates the effect of NF1 diagnosis (i.e., whether it was associated with an increase or decrease in connection strength). In the NF1 group, relative to controls, there was stronger (i.e., more inhibitory) intrinsic (self‐) connectivity in left dlPFC and IPG. In the left hemisphere, dlPFC had more excitatory connections to vlPFC, IPG had more inhibitory connections to dlPFC, SPG and vlPFC, and vlPFC had more excitatory connections to dlPFC and IPG. Within the right hemisphere, dlPFC had more excitatory connections to SPG, SPG had more excitatory connections to IPG, and IPG had more inhibitory connections to vlPFC and vlPFC had more inhibitory connections to dlPFC. Across hemispheres, left dlPFC projected more excitatory connections to right dlPFC, left IPG had more inhibitory connections to right IPG, and left SPEC showed more inhibitory connections to right SPG, while right SPG projected back more excitatory connections to left SPG.

#### 3.3.2 Modulatory Connectivity

**FIGURE 5:**
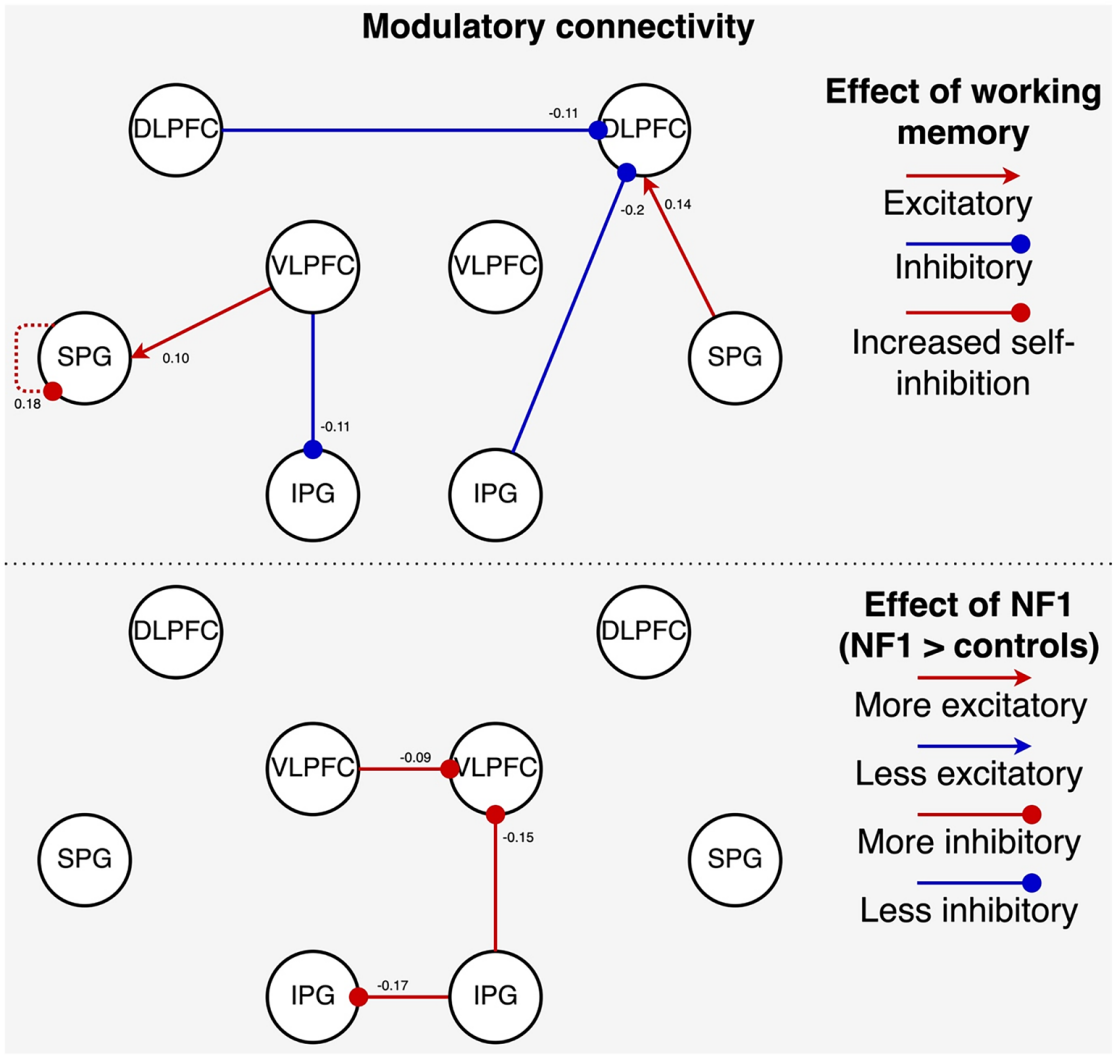
Modulatory effect of working memory on effective connectivity. The top panel shows connections common to both groups, while the bottom panel shows group differences (NF1 > controls). All displayed connections exceed the 95% posterior probability threshold and have strong evidence of being included in the model. In the top graph, red connections ending in an arrowhead indicate excitatory connections, and blue connections ending with a dot indicate inhibitory connections. In the bottom panel, red colour indicates that connections are stronger in NF1, and blue indicates that connections are weaker in NF1. For intrinsic (self‐) connections, ‘stronger’ indicates greater self‐inhibition.

Figure 5 illustrates common (i.e., shared) connection strength, and the bottom graph illustrates the effect of NF1 diagnosis (i.e., whether it was associated with an increase or decrease in connection strength) on the modulatory effect of working memory (B‐matrix). Again, here we focus on strong evidence for group differences. In the NF1 group, relative to controls, working memory evoked more inhibitory connections from right IPG to both left IPG and right vlPFC, and from left vlPFC to right vlPFC.

#### 3.3.3 Hypothesis Testing

BMR was conducted to test the hypothesis that differences in extrinsic modulatory connections were driven by intrinsic (self‐) connectivity (B‐matrix diagonal elements). The winning model, according to BMC, had a posterior probability (*p*_post_) of 0.0352. There was weak evidence to suggest that during working memory, NF1 participants have more inhibitory condition‐related self‐connections in left vlPFC (*p*_post_ = 0.6, expected value of the parameter (Ep) = 0.6), and less inhibitory self‐connections in left dlPFC (*p*_post_ = 0.56, Ep = −0.091), left SPG (*p*_post_ = 0.51, Ep = −0.075) and right IPG (*p*_post_ = 0.66, Ep = −0.120). The parameters of these connections were predictive of NF1 diagnosis; the correlation coefficient between true and predictive scores was 0.34 (*p* = 0.002).

## 4 Discussion

In this work, we investigated whether NF1 participants have altered excitatory and inhibitory signalling in the frontoparietal network that may explain their reduced BOLD activity during verbal working memory. We implemented DCM to characterise the self‐connectivity of regions that show reduced working memory effects in the NF1 group compared to the neurotypical control group. First, we observed that endogenous intrinsic (self‐) connectivity (A‐matrix) of left dlPFC and IPG was increased in NF1, suggesting reduced overall sensitivity of these regions to cognitive inputs. Second, we found that group differences during working memory (B‐matrix) are driven by increased self‐connectivity of left vlPFC and decreased self‐connectivity of left dlPFC, left SPG and right IPG. During cross‐validation, these connectivity patterns were predictive of NF1 diagnosis. Together, these findings suggest that NF1 participants have less sensitivity to task‐related inputs and additionally may be less able to adapt to the task demands of working memory.

To study working memory differences, we selected frontal and parietal regions that showed activity related to working memory in both groups. A meta‐analysis has related the activity of these regions to identity monitoring of verbal stimuli (Owen et al. 2005). In our analysis, these regions showed reduced BOLD activity in response to verbal working memory demands in NF1 participants, as compared to neurotypical controls. This finding of hypoactivity echoes similar findings from previous NF1 literature related to visuospatial working memory (Ibrahim et al. 2017; Shilyansky et al. 2010), and this suggests that hypoactivity may be a consistent feature across cognitive domains. We followed this pattern with DCM analysis, aiming to explore how the neuronal populations within these regions respond to cognitive inputs and specifically to working memory demands, while Supporting Information 1 presents the DCM analysis of regions of shared activity (Seghier et al. 2010).

First, we focused on the analysis of DCM's A‐matrix, which reflects the endogenous or exogenous connectivity. This revealed that NF1 participants had stronger self‐connectivity in the left hemisphere dlPFC and IPG. In DCM, self‐connectivity can be understood as the sensitivity of the region to inputs, where stronger self‐connectivity suggests more inhibition of input‐related signals (Snyder et al. 2021). Therefore, our results suggest the NF1 group has less sensitivity to the N‐back task overall in the parts of the left hemisphere of the frontoparietal network. This altered endogenous connectivity pattern may reflect a compensatory mechanism that allows NF1 participants to perform the N‐back task at the same level as neurotypical controls. This interpretation is consistent with the neural efficiency hypothesis that neurodivergent individuals need to compensate/adapt to their condition through greater neural engagement for equivalent performance (Neubauer and Fink 2009). However, we note that the activation analysis was not conducted to reveal additional recruitment in NF1 participants.

However, Poldrack (2015) raises an important point that observed differences may be indicative of different cognitive processes, different neural computations, differences in neural firing intensity or duration, or true metabolic efficiency differences. Here, our implementation of DCM becomes particularly useful in addressing this ambiguity because it estimates both endogenous and modulatory connectivity. Modulatory connectivity showed that working memory evoked less inhibitory intrinsic (self‐) connectivity in left dlPFC, left SPG and right IPG, but more inhibitory intrinsic (self‐) connectivity in left vlPFC compared to controls. We speculate that this may occur due to high use of neural resources at baseline, which could limit the system's capacity to adapt further to more demanding tasks. Future research should investigate this ceiling effect in neural adaptation by investigating if it can also be found in other executive functions, such as inhibitory control or task switching. The finding that NF1 participants showed reduced modulatory self‐connectivity in left dlPFC suggests that NF1 participants have difficulty modulating their neural activity in response to working memory demands. This may indicate a compromised phonological rehearsal process during working memory because previous research indicates that there is left‐lateralised specialisation of phonological rehearsal during verbal working memory (Crottaz‐Herbette et al. 2004; Emch et al. 2019; Walter et al. 2003). The reduced inhibitory responses in left dlPFC may indicate reduced engagement of these phonological rehearsal processes in adolescents with NF1, which is consistent with previous reports of compromised phonological processing and memory in this population (Arnold et al. 2018; Baudou et al. 2020; Cutting and Levine 2010).

We can further make sense of these differences in cognitive processing between NF1 and controls by considering the cognitive role of individual regions in the fronto‐parietal network. We found that the left vlPFC had stronger inhibitory intrinsic (self‐) connectivity in the NF1 group than in the control group. This region has been related to inhibitory control, attention regulation and filtering of distractors (Aron et al. 2014; Hampshire et al. 2010; Shanmugan et al. 2016). Stronger self‐connectivity of the left vlPFC suggests that it is less sensitive to inputs; therefore, it attenuates more stimuli. In the context of this, we can hypothesize that the NF1 participants stayed more ‘on‐line’ with the presented stimuli, and by extension, it is possible that they committed fewer items to their memory. In contrast, the left dlPFC, left SPG and right IPG were less inhibited (i.e., more driven by the inputs). Individual studies and meta‐analyses show that the dlPFC is involved with executive function, working memory, cognitive control and attention (Jimura et al. 2018; Kim et al. 2015; Osaka et al. 2003; Owen et al. 2005; Rottschy et al. 2012; Vartanian et al. 2013; Wager and Smith 2003; Yaple et al. 2019). Specifically, Huey et al. (2016) proposed that the left dlPFC is involved with the monitoring of individual stimuli or events. Importantly, working memory deficits in individuals with NF1 have been linked to hypoactivity of the left dlPFC (Ibrahim et al. 2017; Shilyansky et al. 2010). Here, we further add to these results the finding that this hypoactivity is related to disrupted inhibitory activity in the region. Future research should focus on investigating these possibilities by correlating working memory performance with performance on tasks dedicated to measuring NF1 participants’ phonological processing, their ability to filter out distractor stimuli and their ability to commit novel items to memory. Among the parietal regions, the left SPG and right IPG were less inhibited. The SPG contributes to working memory tasks requiring manipulation, rather than maintenance and retrieval processes, suggesting its critical role in sequence‐related processing (Koenigs et al. 2009), while the right IPG is more closely associated with retrieval of visuo‐spatial memory (Gray et al. 2020). Their increased sensitivity to working memory inputs in NF1 may reflect disrupted maintenance and over‐responsiveness to new stimuli via increased updating. As such, the NF1 participants may be more prone to interference from new stimuli and find it difficult to maintain a stable representation of the stimulus sequence.

The present work also carries important implications for research focusing on nonpharmacological interventions for NF1. Previous research demonstrated that noninvasive brain stimulation (NIBS) can be used to reduce GABA neurotransmitter concentration in left dlPFC (Garg et al. 2022). Our subsequent work employed the DCM method to understand how NIBS changed the dynamics of these brain regions (Litwińczuk et al. 2025). We showed that after NIBS, less dlPFC GABA was associated with less inhibition of left dlPFC throughout the N‐back task (i.e., weaker intrinsic endogenous connectivity). The present results from this investigation comparing DCM of the NF1 group to the control group add further insight to our understanding of the effects of NIBS; we now see that NIBS has addressed precisely the mechanisms underlying an atypical response in NF1 (i.e., increased intrinsic endogenous connectivity). We also add the new and important finding that NF1 affects inhibitory activity in multiple regions of the frontoparietal network (bilateral vlPFC and left dlPFC). These regions may constitute suitable stimulation sites in future NIBS research. In particular, it will be important to determine what NIBS protocols should be implemented (e.g., optimised electrode placements, use of transcranial direct current stimulation or transcranial alternating current stimulation).

It is important to understand this discussion in the context of the methodological limitations of our analysis. The relatively small sample size of the control group (26) compared to the NF1 group (43) may have reduced the statistical power for detecting group differences. However, this sample size is consistent with sample size recommendations from published DCM work of N‐back task, which demonstrated that samples of 20–27 participants per group can provide sufficient sensitivity to detect group differences in DCM parameters (Goulden et al. 2012). Additionally, the 2‐back condition produced ceiling effects in both NF1 and control groups, limiting variability in behavioural performance measures. Consequently, we were unable to examine brain‐behaviour relationships. Future studies could employ more challenging working memory conditions and a variety of executive function tasks to better characterise the relationship between connectivity parameters and task performance. Next, the DCM analysis produces models of interactions between a set of brain regions. This means that other neuronal populations, outside the set of chosen VOIs, also likely contribute to the dynamics of the regions we choose to study. In other words, the hypoactivity reported here is limited to the context of processes of frontoparietal networks. To illustrate, we explored in this analysis how working memory inputs enter, how they are maintained and manipulated, but we cannot comment on other processes such as inhibitory control, attentional processing, early visual processes or motor processing. Each of these components is a subject of its own study and offers opportunities for future research. Similarly, due to our cross‐sectional design, we cannot draw any conclusions about the developmental trajectories, which may reveal the maturation of inhibitory signalling mechanisms. Additionally, it is important to note that NF1 participants were not off their regular medications, which may influence their cognitive processing. We did not include either medication status in the PEB analysis, nor comorbid diagnosis with other neurodevelopmental conditions. We also did not collect demographic information about participants' handedness or their education level, which could have aided the interpretation of hemispheric differences and further improved developmental comparability between groups. These covariates can impact individual differences in cognition and brain function; therefore, future work should explore how they contribute to neurodivergent developmental pathways.

Overall, this work has demonstrated that NF1 participants have increased endogenous intrinsic (self‐) connectivity in left dlPFC and left IPG during the N‐back task, suggesting increased inhibition and therefore reduced sensitivity to inputs. Additionally, NF1 participants showed reduced inhibitory responses in left dlPFC and SPG during working memory demands but increased modulatory self‐connectivity in left vlPFC. These findings suggest that NF1 participants have less sensitivity to task‐related inputs and additionally may be less able to adapt to the task demands of working memory.

## Funding

This work was supported by the Medical Research Council (MR/X005267/1), Neurofibromatosis Therapeutic Acceleration Program and Manchester Biomedical Research Centre (NIHR203308).

## Disclosure

Participant Inclusivity—Statement 2.

2. My article reports human subjects. Recruitment meets scientific requirements & HBMs expectation of inclusivity.

## Ethics Statement

Ethics approval for the study was obtained from the North West‐Greater Manchester South Research Ethics Committee (reference: 18/NW/0762). Written informed consent was obtained from the parents and older adolescent participants and assent was obtained from the younger participants.

## Conflicts of Interest

The authors declare no conflicts of interest.

## Supporting information


**Supporting Information: 1** DCM models of shared activation.

**Supporting Information: 2**. hbm70464‐sup‐0002‐Supinfo2.docx

**Supporting Information: 3**. hbm70464‐sup‐0003‐Supinfo3.docx

## Data Availability

The NF1 group's data have been deposited on the Sage Bionetworks data repository https://www.synapse.org/. Approved researchers can request to obtain the data that are subject to data sharing agreements. Codes for data processing and analysis are available at https://github.com/MCLit/NF1‐DCM‐WM.
