# Supplementary material for: Disrupted Frontoparietal Dynamics in Neurofibromatosis Type 1: Reduced Sensitivity and Atypical Modulation During Working Memory
Source: Hum Brain Mapp. 2026 Feb 3;47(2):e70464. doi: 10.1002/hbm.70464 (PMC12865864; doi:10.1002/hbm.70464)
Supplement: Supplementary file 1 — Supporting Information: 1 DCM models of shared activation. [file HBM-47-e70464-s003.docx]

**Supplementary Material 1 – DCM models of shared activation**

- 1. **Volumes of Interest**

Eight volumes of interest (VOIs) were defined in bilateral superior parietal gyrus (SPG), bilateral inferior parietal gyrus (IPG), bilateral ventrolateral prefrontal cortex (VLPFC), bilateral dorsolateral prefrontal cortex (DLPFC). Their centre coordinates were placed based on the peak activation related to the effect of working memory in both groups (NF1 and control average contrast). Supplementary Table 1.1 lists the MNI coordinates where VOI spheres were placed, and Supplementary Figure 1 illustrates their placement and shape. VOIs were defined as spheres with radius of 6mm, and they were additionally masked by the average mask of effect of working memory, derived from the mass univariate analysis. This ensured that models reflect only the neuronal activity related to shared working memory effects.

| Supplementary Table 1.1 The MNI coordinates of the centre of the VOI spheres. | | |
| --- | --- | --- |
|  | Left | Right |
| SPG | [-24, -64, 50] | [28, -58, 48] |
| vlPFC | [-44, 16, 2] | [44, 18, -2] |
| dlPFC | [-42, 24, 28] | [46, 32, 28] |
| IPG | [-34, -44, 40] | [36, -46, 38] |


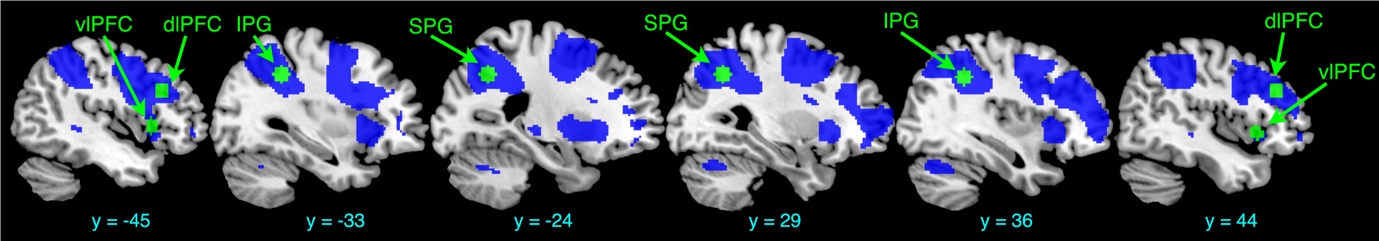


Supplementary Figure 1 A binarized overlay of clusters generated by mass univariate analysis showing regions where both the NF1 and the control group have working memory related activation. Green voxels illustrate the VOIs. The cyan y-coordinates are the MNI coordinates of the sagittal slices.

- 1. **Effective Connectivity**
     1. **Average Connectivity**

The top graph in Supplementary Figure 2 illustrates the shared connection strength and bottom graph illustrates effect of NF1 diagnosis. Both the figure and the text focus on strong evidence (posterior probability of including the connection in the model >0.95%).

In the NF1 group, relative to controls, there was stronger intrinsic connectivity (i.e. more inhibitory) in left vlPFC, and weaker intrinsic connectivity (i.e. less inhibitory) in right IPG. Within the left hemisphere, IPG had increased excitatory connections to both dlPFC and vlPFC. Additionally, dlPFC had more inhibitory connections to left SPG and vlPFC projected more inhibitory connections to both dlPFC and IPG. In the right hemisphere, IPG received more inhibitory connections from dlPFC and SPG, yet projected more excitatory connection to vlPFC. Meanwhile, SPG exchanged more inhibitory connections with dlPFC and projected less inhibitory connection to vlPFC. Across hemispheres, right dlPFC projected stronger excitatory connection to right dlPFC, and both left vlPFC and SPG projected stronger excitatory connections to their right hemisphere homologues.


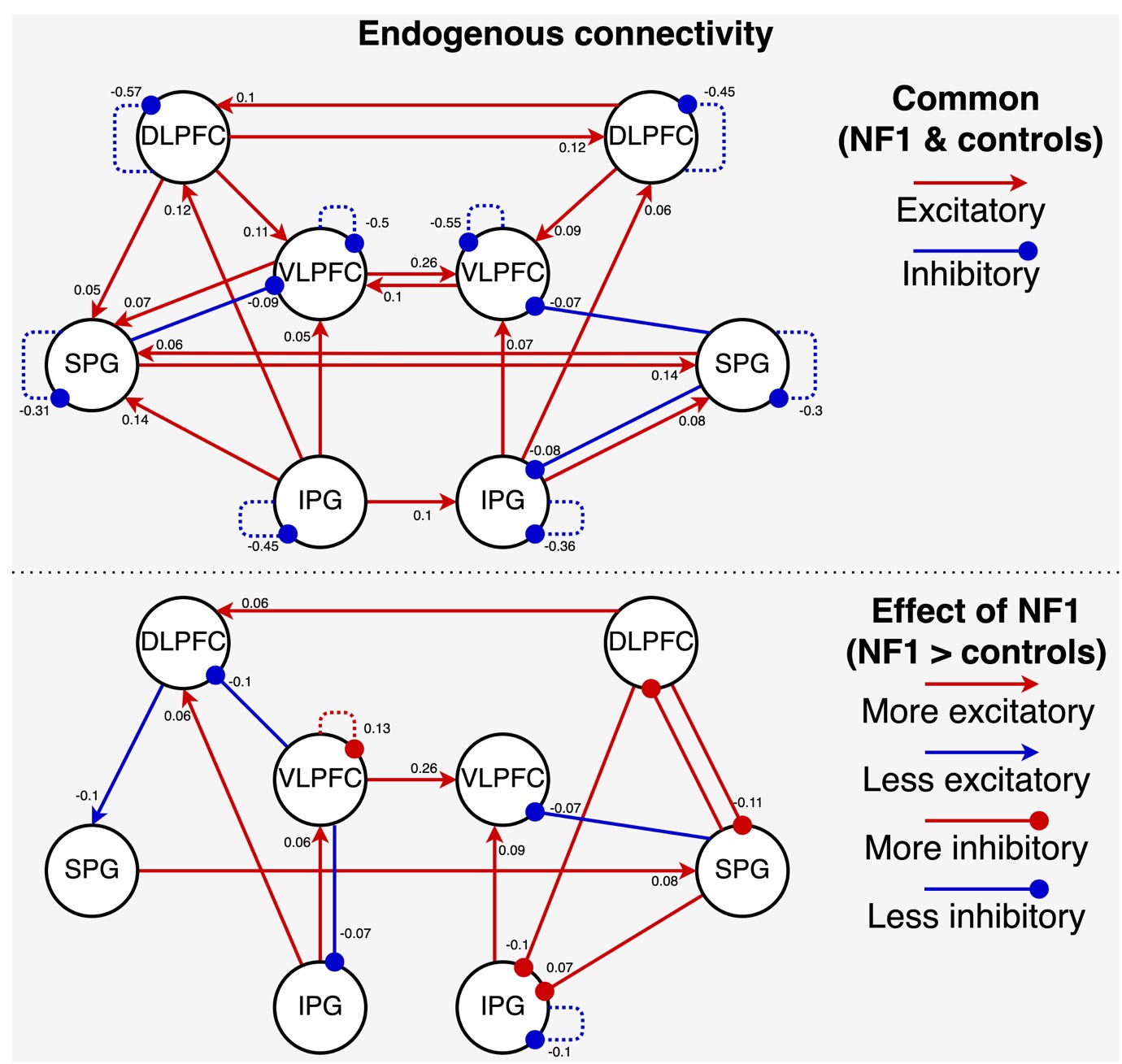


Supplementary Figure 2 The endogenous connectivity during 0-back and 2-back conditions of the N-back task (i.e. A-matrix). Top panel shows connections common to both groups; bottom panel shows group differences (NF1>controls). All connections illustrated here exceed the 95% posterior probability threshold and have strong evidence of being included in the model. In the top graph red indicates excitatory and blue inhibitory influences. In the bottom panel, red connections are stronger in NF1 and blue weaker. For intrinsic (self) connections, "stronger" indicates greater self-inhibition.

- - 1. **Modulatory Connectivity**

The top graph in Supplementary Figure 3 illustrates the shared connection strength and bottom graph illustrates effect of NF1 diagnosis. Both the figure and the text focus on strong evidence (posterior probability of including the connection in the model >0.95%).

In NF1, relative to controls, working memory evoked less inhibitory intrinsic connectivity (self-connectivity) of left vlPFC, and extrinsic connectivity from right dlPFC to right dlPFC to right SPG. In contrast, in NF1 there was more inhibitory connectivity from right IPG to right SPG. Additionally, there was more expiatory connectivity from left SPG to left vlPFC.


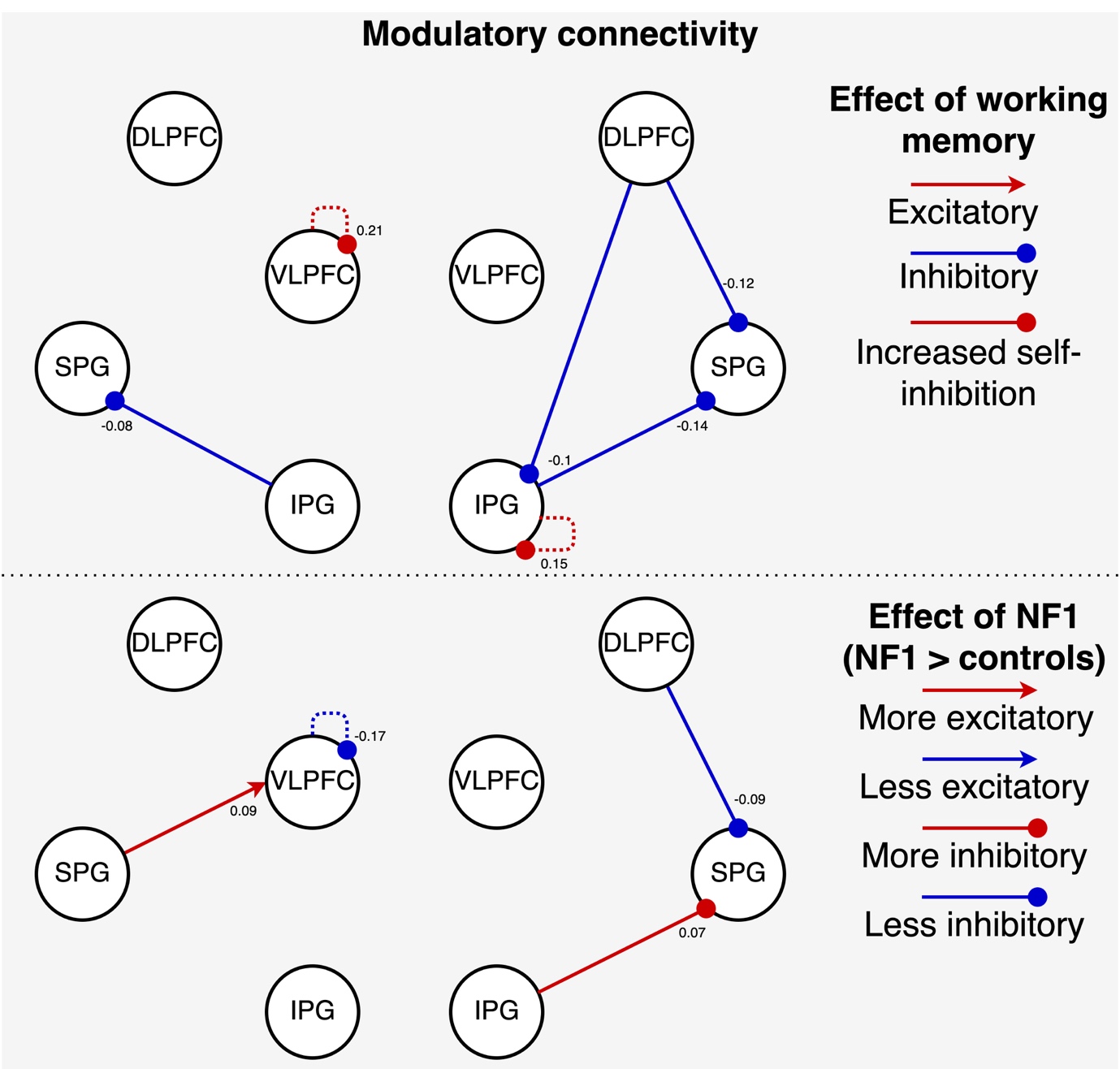


Supplementary Figure 3 Modulatory effect of working memory on effective connectivity (i.e. B-matrix). Top panel shows connections common to both groups; bottom panel shows group differences (NF1>controls). All displayed connections exceed the 95% posterior probability threshold and have strong evidence of being included in the model. In the top panels red indicates excitatory and blue inhibitory influences. In the bottom panel, red connections are stronger in NF1 and blue weaker. For intrinsic (self) connections, "stronger" indicates greater self-inhibition.

- - 1. **Hypothesis testing**

BMR was conducted to test the hypothesis that differences in extrinsic modulatory connections were driven by intrinsic (self-) connectivity (B-matrix diagonal elements). The winning model according to BMC had a posterior probability (*p*_post_) of 0.0352. It suggested that during working memory NF1 participants have more inhibitory condition-related self-connections in left SPG (*p*_post_ = 0.63, expected value of the parameter (Ep) = 0.1), and less-inhibitory self-connections in left vlPFC (*p*_post_ = 0.87, Ep = -0.173). The parameters of these connections were not predictive of NF1 diagnosis; correlation coefficient between true and predictive scores was 0.15 (*p* = 0.103).

- - 1. **Precise parameter values**

Precise parameter values and their posterior probabilities for endogenous and modulatory connectivity, reported in this Supplementary Material 1. VOIs were positioned at peak group activation (controls and NF1 mass univariate contrast).

| Supplementary Table 1.2 The precise values of all connections from the A-matrix, reported in the main manuscript. Ep = expected value of the parameter. Ppost = posterior probability of the parameter. | | | | | |
| --- | --- | --- | --- | --- | --- |
| **Parameter** | **Origin** | **Destination** | **Ep** | **Ppost** | **Ppost > 95%** |
| **Covariate 1: Shared connectivity** | | | | | |
| A(1,1) | left dlPFC | left dlPFC | -0.573 | 1 | * |
| A(2,1) | left dlPFC | right dlPFC | 0.116 | 1 | * |
| A(3,1) | left dlPFC | left IPG | 0 | 0 |  |
| A(5,1) | left dlPFC | left SPG | 0.046 | 1 | * |
| A(7,1) | left dlPFC | left vlPFC | 0.112 | 1 | * |
| A(1,2) | right dlPFC | left dlPFC | 0.097 | 1 | * |
| A(2,2) | right dlPFC | right dlPFC | -0.451 | 1 | * |
| A(4,2) | right dlPFC | right IPG | 0 | 0 |  |
| A(6,2) | right dlPFC | right SPG | 0 | 0 |  |
| A(8,2) | right dlPFC | right vlPFC | 0.09 | 1 | * |
| A(1,3) | left IPG | left dlPFC | 0.123 | 1 | * |
| A(3,3) | left IPG | left IPG | -0.454 | 1 | * |
| A(4,3) | left IPG | right IPG | 0.102 | 1 | * |
| A(5,3) | left IPG | left SPG | 0.137 | 1 | * |
| A(7,3) | left IPG | left vlPFC | 0.051 | 1 | * |
| A(2,4) | right IPG | right dlPFC | 0.061 | 1 | * |
| A(3,4) | right IPG | left IPG | 0 | 0 |  |
| A(4,4) | right IPG | right IPG | -0.36 | 1 | * |
| A(6,4) | right IPG | right SPG | 0.078 | 1 | * |
| A(8,4) | right IPG | right vlPFC | 0.065 | 1 | * |
| A(1,5) | left SPG | left dlPFC | 0 | 0 |  |
| A(3,5) | left SPG | left IPG | 0 | 0 |  |
| A(5,5) | left SPG | left SPG | -0.309 | 1 | * |
| A(6,5) | left SPG | right SPG | 0.141 | 1 | * |
| A(7,5) | left SPG | left vlPFC | -0.086 | 1 | * |
| A(2,6) | right SPG | right dlPFC | 0 | 0 |  |
| A(4,6) | right SPG | right IPG | -0.075 | 1 | * |
| A(5,6) | right SPG | left SPG | 0.061 | 1 | * |
| A(6,6) | right SPG | right SPG | -0.302 | 1 | * |
| A(8,6) | right SPG | right vlPFC | -0.067 | 1 | * |
| A(1,7) | left vlPFC | left dlPFC | 0 | 0 |  |
| A(3,7) | left vlPFC | left IPG | 0 | 0 |  |
| A(5,7) | left vlPFC | left SPG | 0.069 | 1 | * |
| A(7,7) | left vlPFC | left vlPFC | -0.506 | 1 | * |
| A(8,7) | left vlPFC | right vlPFC | 0.256 | 1 | * |
| A(2,8) | right vlPFC | right dlPFC | 0 | 0 |  |
| A(4,8) | right vlPFC | right IPG | 0 | 0 |  |
| A(6,8) | right vlPFC | right SPG | 0 | 0 |  |
| A(7,8) | right vlPFC | left vlPFC | 0.096 | 1 | * |
| A(8,8) | right vlPFC | right vlPFC | -0.545 | 1 | * |
| **Covariate 2: Effect of NF1 diagnosis** | | | | | |
| A(1,1) | left dlPFC | left dlPFC | 0 | 0 |  |
| A(2,1) | left dlPFC | right dlPFC | 0 | 0 |  |
| A(3,1) | left dlPFC | left IPG | 0 | 0 |  |
| A(5,1) | left dlPFC | left SPG | -0.103 | 1 | * |
| A(7,1) | left dlPFC | left vlPFC | 0 | 0 |  |
| A(1,2) | right dlPFC | left dlPFC | 0.059 | 1 | * |
| A(2,2) | right dlPFC | right dlPFC | 0 | 0 |  |
| A(4,2) | right dlPFC | right IPG | -0.098 | 1 | * |
| A(6,2) | right dlPFC | right SPG | -0.11 | 1 | * |
| A(8,2) | right dlPFC | right vlPFC | 0 | 0 |  |
| A(1,3) | left IPG | left dlPFC | 0.062 | 1 | * |
| A(3,3) | left IPG | left IPG | 0 | 0 |  |
| A(4,3) | left IPG | right IPG | 0 | 0 |  |
| A(5,3) | left IPG | left SPG | 0 | 0 |  |
| A(7,3) | left IPG | left vlPFC | 0.062 | 1 | * |
| A(2,4) | right IPG | right dlPFC | 0 | 0 |  |
| A(3,4) | right IPG | left IPG | 0 | 0 |  |
| A(4,4) | right IPG | right IPG | -0.1 | 1 | * |
| A(6,4) | right IPG | right SPG | 0 | 0 |  |
| A(8,4) | right IPG | right vlPFC | 0.088 | 1 | * |
| A(1,5) | left SPG | left dlPFC | 0 | 0 |  |
| A(3,5) | left SPG | left IPG | 0.017 | 0.49 |  |
| A(5,5) | left SPG | left SPG | -0.037 | 0.55 |  |
| A(6,5) | left SPG | right SPG | 0.081 | 1 | * |
| A(7,5) | left SPG | left vlPFC | 0 | 0 |  |
| A(2,6) | right SPG | right dlPFC | -0.067 | 1 | * |
| A(4,6) | right SPG | right IPG | 0.066 | 1 | * |
| A(5,6) | right SPG | left SPG | 0 | 0 |  |
| A(6,6) | right SPG | right SPG | 0 | 0 |  |
| A(8,6) | right SPG | right vlPFC | 0.029 | 0.54 |  |
| A(1,7) | left vlPFC | left dlPFC | -0.102 | 1 | * |
| A(3,7) | left vlPFC | left IPG | -0.071 | 1 | * |
| A(5,7) | left vlPFC | left SPG | 0 | 0 |  |
| A(7,7) | left vlPFC | left vlPFC | 0.129 | 1 | * |
| A(8,7) | left vlPFC | right vlPFC | 0 | 0 |  |
| A(2,8) | right vlPFC | right dlPFC | 0 | 0 |  |
| A(4,8) | right vlPFC | right IPG | 0 | 0 |  |
| A(6,8) | right vlPFC | right SPG | 0 | 0 |  |
| A(7,8) | right vlPFC | left vlPFC | 0 | 0 |  |
| A(8,8) | right vlPFC | right vlPFC | 0.034 | 0.55 |  |
| **Covariate 3: Age** | | | | | |
| A(1,1) | left dlPFC | left dlPFC | 0 | 0 |  |
| A(2,1) | left dlPFC | right dlPFC | 0 | 0 |  |
| A(3,1) | left dlPFC | left IPG | 0 | 0 |  |
| A(5,1) | left dlPFC | left SPG | 0 | 0 |  |
| A(7,1) | left dlPFC | left vlPFC | 0 | 0 |  |
| A(1,2) | right dlPFC | left dlPFC | -0.03 | 1 | * |
| A(2,2) | right dlPFC | right dlPFC | 0 | 0 |  |
| A(4,2) | right dlPFC | right IPG | 0 | 0 |  |
| A(6,2) | right dlPFC | right SPG | 0 | 0 |  |
| A(8,2) | right dlPFC | right vlPFC | 0 | 0 |  |
| A(1,3) | left IPG | left dlPFC | 0.034 | 1 | * |
| A(3,3) | left IPG | left IPG | 0 | 0 |  |
| A(4,3) | left IPG | right IPG | 0 | 0 |  |
| A(5,3) | left IPG | left SPG | 0 | 0 |  |
| A(7,3) | left IPG | left vlPFC | 0 | 0 |  |
| A(2,4) | right IPG | right dlPFC | 0 | 0 |  |
| A(3,4) | right IPG | left IPG | 0 | 0 |  |
| A(4,4) | right IPG | right IPG | 0 | 0 |  |
| A(6,4) | right IPG | right SPG | -0.03 | 1 | * |
| A(8,4) | right IPG | right vlPFC | -0.029 | 1 | * |
| A(1,5) | left SPG | left dlPFC | 0 | 0 |  |
| A(3,5) | left SPG | left IPG | 0 | 0 |  |
| A(5,5) | left SPG | left SPG | 0 | 0 |  |
| A(6,5) | left SPG | right SPG | 0 | 0 |  |
| A(7,5) | left SPG | left vlPFC | 0.022 | 1 | * |
| A(2,6) | right SPG | right dlPFC | 0 | 0 |  |
| A(4,6) | right SPG | right IPG | -0.024 | 1 | * |
| A(5,6) | right SPG | left SPG | 0 | 0 |  |
| A(6,6) | right SPG | right SPG | -0.021 | 0.61 |  |
| A(8,6) | right SPG | right vlPFC | 0 | 0 |  |
| A(1,7) | left vlPFC | left dlPFC | 0 | 0 |  |
| A(3,7) | left vlPFC | left IPG | 0 | 0 |  |
| A(5,7) | left vlPFC | left SPG | 0 | 0 |  |
| A(7,7) | left vlPFC | left vlPFC | 0.046 | 1 | * |
| A(8,7) | left vlPFC | right vlPFC | 0 | 0 |  |
| A(2,8) | right vlPFC | right dlPFC | 0 | 0 |  |
| A(4,8) | right vlPFC | right IPG | 0 | 0 |  |
| A(6,8) | right vlPFC | right SPG | -0.021 | 1 | * |
| A(7,8) | right vlPFC | left vlPFC | 0 | 0 |  |
| A(8,8) | right vlPFC | right vlPFC | 0 | 0 |  |
| **Covariate 4: Sex (1=male)** | | | | | |
| A(1,1) | left dlPFC | left dlPFC | -0.193 | 1 | * |
| A(2,1) | left dlPFC | right dlPFC | 0 | 0 |  |
| A(3,1) | left dlPFC | left IPG | 0.148 | 1 | * |
| A(5,1) | left dlPFC | left SPG | 0.24 | 1 | * |
| A(7,1) | left dlPFC | left vlPFC | 0 | 0 |  |
| A(1,2) | right dlPFC | left dlPFC | 0 | 0 |  |
| A(2,2) | right dlPFC | right dlPFC | 0 | 0 |  |
| A(4,2) | right dlPFC | right IPG | 0.132 | 1 | * |
| A(6,2) | right dlPFC | right SPG | 0.277 | 1 | * |
| A(8,2) | right dlPFC | right vlPFC | 0.171 | 1 | * |
| A(1,3) | left IPG | left dlPFC | 0 | 0 |  |
| A(3,3) | left IPG | left IPG | 0.21 | 1 | * |
| A(4,3) | left IPG | right IPG | 0 | 0 |  |
| A(5,3) | left IPG | left SPG | -0.169 | 1 | * |
| A(7,3) | left IPG | left vlPFC | 0 | 0 |  |
| A(2,4) | right IPG | right dlPFC | 0 | 0 |  |
| A(3,4) | right IPG | left IPG | -0.047 | 0.53 |  |
| A(4,4) | right IPG | right IPG | 0.32 | 1 | * |
| A(6,4) | right IPG | right SPG | 0 | 0 |  |
| A(8,4) | right IPG | right vlPFC | 0 | 0 |  |
| A(1,5) | left SPG | left dlPFC | 0.121 | 1 | * |
| A(3,5) | left SPG | left IPG | 0 | 0 |  |
| A(5,5) | left SPG | left SPG | 0.08 | 0.56 |  |
| A(6,5) | left SPG | right SPG | -0.114 | 1 | * |
| A(7,5) | left SPG | left vlPFC | 0 | 0 |  |
| A(2,6) | right SPG | right dlPFC | 0 | 0 |  |
| A(4,6) | right SPG | right IPG | 0 | 0 |  |
| A(5,6) | right SPG | left SPG | 0 | 0 |  |
| A(6,6) | right SPG | right SPG | 0.198 | 1 | * |
| A(8,6) | right SPG | right vlPFC | 0 | 0 |  |
| A(1,7) | left vlPFC | left dlPFC | 0 | 0 |  |
| A(3,7) | left vlPFC | left IPG | 0.095 | 1 | * |
| A(5,7) | left vlPFC | left SPG | -0.117 | 1 | * |
| A(7,7) | left vlPFC | left vlPFC | 0 | 0 |  |
| A(8,7) | left vlPFC | right vlPFC | -0.105 | 1 | * |
| A(2,8) | right vlPFC | right dlPFC | -0.045 | 0.6 |  |
| A(4,8) | right vlPFC | right IPG | 0 | 0 |  |
| A(6,8) | right vlPFC | right SPG | -0.132 | 1 | * |
| A(7,8) | right vlPFC | left vlPFC | -0.132 | 1 | * |
| A(8,8) | right vlPFC | right vlPFC | 0 | 0 |  |

| Supplementary Table 1.3 The precise values of all connections from the A-matrix, reported in the main manuscript. Ep = expected value of the parameter. Ppost = posterior probability of the parameter. | | | | | |
| --- | --- | --- | --- | --- | --- |
| **Parameter** | **Origin** | **Destination** | **Ep** | **Ppost** | **Ppost > 95%** |
| **Covariate 1: Shared connectivity** | | | | | |
| B(1,1) | left dlPFC | left dlPFC | -0.066 | 0.79 |  |
| B(2,1) | left dlPFC | right dlPFC | -0.023 | 0.7 |  |
| B(3,1) | left dlPFC | left IPG | -0.071 | 0.95 |  |
| B(5,1) | left dlPFC | left SPG | -0.043 | 0.83 |  |
| B(7,1) | left dlPFC | left vlPFC | -0.01 | 0.58 |  |
| B(1,2) | right dlPFC | left dlPFC | -0.062 | 0.9 |  |
| B(2,2) | right dlPFC | right dlPFC | 0.128 | 0.93 |  |
| B(4,2) | right dlPFC | right IPG | -0.097 | 0.98 | * |
| B(6,2) | right dlPFC | right SPG | -0.115 | 0.99 | * |
| B(8,2) | right dlPFC | right vlPFC | -0.041 | 0.79 |  |
| B(1,3) | left IPG | left dlPFC | -0.004 | 0.54 |  |
| B(3,3) | left IPG | left IPG | 0.023 | 0.64 |  |
| B(4,3) | left IPG | right IPG | 0.002 | 0.52 |  |
| B(5,3) | left IPG | left SPG | -0.08 | 0.96 | * |
| B(7,3) | left IPG | left vlPFC | 0.074 | 0.93 |  |
| B(2,4) | right IPG | right dlPFC | -0.004 | 0.53 |  |
| B(3,4) | right IPG | left IPG | -0.017 | 0.64 |  |
| B(4,4) | right IPG | right IPG | 0.148 | 0.96 | * |
| B(6,4) | right IPG | right SPG | -0.136 | 1 | * |
| B(8,4) | right IPG | right vlPFC | -0.002 | 0.51 |  |
| B(1,5) | left SPG | left dlPFC | -0.028 | 0.7 |  |
| B(3,5) | left SPG | left IPG | -0.01 | 0.58 |  |
| B(5,5) | left SPG | left SPG | 0.051 | 0.75 |  |
| B(6,5) | left SPG | right SPG | -0.008 | 0.57 |  |
| B(7,5) | left SPG | left vlPFC | 0.028 | 0.72 |  |
| B(2,6) | right SPG | right dlPFC | -0.007 | 0.56 |  |
| B(4,6) | right SPG | right IPG | 0.035 | 0.75 |  |
| B(5,6) | right SPG | left SPG | -0.019 | 0.66 |  |
| B(6,6) | right SPG | right SPG | 0.008 | 0.54 |  |
| B(8,6) | right SPG | right vlPFC | 0.048 | 0.81 |  |
| B(1,7) | left vlPFC | left dlPFC | 0.066 | 0.92 |  |
| B(3,7) | left vlPFC | left IPG | 0.068 | 0.94 |  |
| B(5,7) | left vlPFC | left SPG | 0.015 | 0.63 |  |
| B(7,7) | left vlPFC | left vlPFC | 0.214 | 1 | * |
| B(8,7) | left vlPFC | right vlPFC | 0.005 | 0.54 |  |
| B(2,8) | right vlPFC | right dlPFC | 0.018 | 0.66 |  |
| B(4,8) | right vlPFC | right IPG | 0.035 | 0.8 |  |
| B(6,8) | right vlPFC | right SPG | -0.002 | 0.52 |  |
| B(7,8) | right vlPFC | left vlPFC | 0.027 | 0.74 |  |
| B(8,8) | right vlPFC | right vlPFC | 0.001 | 0.51 |  |
| **Covariate 2: Effect of NF1 diagnosis** | | | | | |
| B(1,1) | left dlPFC | left dlPFC | -0.026 | 0.63 |  |
| B(2,1) | left dlPFC | right dlPFC | 0.025 | 0.71 |  |
| B(3,1) | left dlPFC | left IPG | 0.059 | 0.9 |  |
| B(5,1) | left dlPFC | left SPG | -0.003 | 0.52 |  |
| B(7,1) | left dlPFC | left vlPFC | -0.057 | 0.88 |  |
| B(1,2) | right dlPFC | left dlPFC | -0.034 | 0.75 |  |
| B(2,2) | right dlPFC | right dlPFC | 0.026 | 0.64 |  |
| B(4,2) | right dlPFC | right IPG | -0.025 | 0.7 |  |
| B(6,2) | right dlPFC | right SPG | -0.093 | 0.98 | * |
| B(8,2) | right dlPFC | right vlPFC | -0.004 | 0.53 |  |
| B(1,3) | left IPG | left dlPFC | -0.052 | 0.88 |  |
| B(3,3) | left IPG | left IPG | 0.042 | 0.73 |  |
| B(4,3) | left IPG | right IPG | -0.019 | 0.64 |  |
| B(5,3) | left IPG | left SPG | -0.002 | 0.51 |  |
| B(7,3) | left IPG | left vlPFC | -0.01 | 0.58 |  |
| B(2,4) | right IPG | right dlPFC | 0.005 | 0.54 |  |
| B(3,4) | right IPG | left IPG | 0.026 | 0.7 |  |
| B(4,4) | right IPG | right IPG | 0.062 | 0.78 |  |
| B(6,4) | right IPG | right SPG | 0.076 | 0.97 | * |
| B(8,4) | right IPG | right vlPFC | 0.043 | 0.79 |  |
| B(1,5) | left SPG | left dlPFC | 0.062 | 0.9 |  |
| B(3,5) | left SPG | left IPG | -0.012 | 0.6 |  |
| B(5,5) | left SPG | left SPG | 0.101 | 0.89 |  |
| B(6,5) | left SPG | right SPG | -0.01 | 0.59 |  |
| B(7,5) | left SPG | left vlPFC | 0.094 | 0.97 | * |
| B(2,6) | right SPG | right dlPFC | -0.066 | 0.9 |  |
| B(4,6) | right SPG | right IPG | 0.007 | 0.55 |  |
| B(5,6) | right SPG | left SPG | 0.037 | 0.78 |  |
| B(6,6) | right SPG | right SPG | -0.004 | 0.52 |  |
| B(8,6) | right SPG | right vlPFC | -0.049 | 0.81 |  |
| B(1,7) | left vlPFC | left dlPFC | 0.005 | 0.54 |  |
| B(3,7) | left vlPFC | left IPG | -0.049 | 0.85 |  |
| B(5,7) | left vlPFC | left SPG | 0.024 | 0.7 |  |
| B(7,7) | left vlPFC | left vlPFC | -0.173 | 0.98 | * |
| B(8,7) | left vlPFC | right vlPFC | -0.007 | 0.56 |  |
| B(2,8) | right vlPFC | right dlPFC | 0.021 | 0.69 |  |
| B(4,8) | right vlPFC | right IPG | -0.026 | 0.73 |  |
| B(6,8) | right vlPFC | right SPG | 0.007 | 0.56 |  |
| B(7,8) | right vlPFC | left vlPFC | -0.055 | 0.88 |  |
| B(8,8) | right vlPFC | right vlPFC | 0.035 | 0.69 |  |
| **Covariate 3: Age** | | | | | |
| B(1,1) | left dlPFC | left dlPFC | 0.065 | 0.96 | * |
| B(2,1) | left dlPFC | right dlPFC | -0.015 | 0.8 |  |
| B(3,1) | left dlPFC | left IPG | -0.023 | 0.89 |  |
| B(5,1) | left dlPFC | left SPG | 0.009 | 0.67 |  |
| B(7,1) | left dlPFC | left vlPFC | -0.024 | 0.88 |  |
| B(1,2) | right dlPFC | left dlPFC | 0.009 | 0.66 |  |
| B(2,2) | right dlPFC | right dlPFC | -0.01 | 0.6 |  |
| B(4,2) | right dlPFC | right IPG | -0.012 | 0.72 |  |
| B(6,2) | right dlPFC | right SPG | -0.01 | 0.68 |  |
| B(8,2) | right dlPFC | right vlPFC | 0.007 | 0.61 |  |
| B(1,3) | left IPG | left dlPFC | 0.027 | 0.92 |  |
| B(3,3) | left IPG | left IPG | -0.022 | 0.75 |  |
| B(4,3) | left IPG | right IPG | -0.014 | 0.72 |  |
| B(5,3) | left IPG | left SPG | -0.005 | 0.6 |  |
| B(7,3) | left IPG | left vlPFC | 0.022 | 0.84 |  |
| B(2,4) | right IPG | right dlPFC | 0.001 | 0.52 |  |
| B(3,4) | right IPG | left IPG | -0.003 | 0.55 |  |
| B(4,4) | right IPG | right IPG | -0.045 | 0.9 |  |
| B(6,4) | right IPG | right SPG | -0.003 | 0.56 |  |
| B(8,4) | right IPG | right vlPFC | -0.014 | 0.72 |  |
| B(1,5) | left SPG | left dlPFC | 0.008 | 0.64 |  |
| B(3,5) | left SPG | left IPG | 0.01 | 0.68 |  |
| B(5,5) | left SPG | left SPG | 0.03 | 0.82 |  |
| B(6,5) | left SPG | right SPG | 0.025 | 0.91 |  |
| B(7,5) | left SPG | left vlPFC | -0.031 | 0.92 |  |
| B(2,6) | right SPG | right dlPFC | 0.019 | 0.79 |  |
| B(4,6) | right SPG | right IPG | -0.004 | 0.57 |  |
| B(5,6) | right SPG | left SPG | 0.011 | 0.71 |  |
| B(6,6) | right SPG | right SPG | 0.005 | 0.56 |  |
| B(8,6) | right SPG | right vlPFC | -0.029 | 0.87 |  |
| B(1,7) | left vlPFC | left dlPFC | -0.001 | 0.52 |  |
| B(3,7) | left vlPFC | left IPG | 0.015 | 0.79 |  |
| B(5,7) | left vlPFC | left SPG | 0.003 | 0.56 |  |
| B(7,7) | left vlPFC | left vlPFC | 0.006 | 0.57 |  |
| B(8,7) | left vlPFC | right vlPFC | 0.01 | 0.69 |  |
| B(2,8) | right vlPFC | right dlPFC | -0.005 | 0.6 |  |
| B(4,8) | right vlPFC | right IPG | 0.031 | 0.96 | * |
| B(6,8) | right vlPFC | right SPG | -0.013 | 0.74 |  |
| B(7,8) | right vlPFC | left vlPFC | 0.023 | 0.88 |  |
| B(8,8) | right vlPFC | right vlPFC | -0.008 | 0.59 |  |
| **Covariate 4: Sex (1=male)** | | | | | |
| B(1,1) | left dlPFC | left dlPFC | -0.195 | 0.88 |  |
| B(2,1) | left dlPFC | right dlPFC | 0.071 | 0.79 |  |
| B(3,1) | left dlPFC | left IPG | -0.176 | 0.97 | * |
| B(5,1) | left dlPFC | left SPG | 0.103 | 0.87 |  |
| B(7,1) | left dlPFC | left vlPFC | 0.094 | 0.83 |  |
| B(1,2) | right dlPFC | left dlPFC | -0.013 | 0.55 |  |
| B(2,2) | right dlPFC | right dlPFC | -0.029 | 0.56 |  |
| B(4,2) | right dlPFC | right IPG | 0.118 | 0.9 |  |
| B(6,2) | right dlPFC | right SPG | -0.021 | 0.59 |  |
| B(8,2) | right dlPFC | right vlPFC | -0.102 | 0.84 |  |
| B(1,3) | left IPG | left dlPFC | 0.147 | 0.95 |  |
| B(3,3) | left IPG | left IPG | -0.143 | 0.84 |  |
| B(4,3) | left IPG | right IPG | -0.004 | 0.52 |  |
| B(5,3) | left IPG | left SPG | 0.046 | 0.69 |  |
| B(7,3) | left IPG | left vlPFC | 0.027 | 0.61 |  |
| B(2,4) | right IPG | right dlPFC | 0.074 | 0.77 |  |
| B(3,4) | right IPG | left IPG | 0.079 | 0.8 |  |
| B(4,4) | right IPG | right IPG | -0.074 | 0.68 |  |
| B(6,4) | right IPG | right SPG | 0.083 | 0.84 |  |
| B(8,4) | right IPG | right vlPFC | -0.105 | 0.85 |  |
| B(1,5) | left SPG | left dlPFC | -0.006 | 0.52 |  |
| B(3,5) | left SPG | left IPG | 0.068 | 0.76 |  |
| B(5,5) | left SPG | left SPG | -0.036 | 0.59 |  |
| B(6,5) | left SPG | right SPG | 0.096 | 0.87 |  |
| B(7,5) | left SPG | left vlPFC | -0.042 | 0.66 |  |
| B(2,6) | right SPG | right dlPFC | -0.001 | 0.5 |  |
| B(4,6) | right SPG | right IPG | 0.039 | 0.64 |  |
| B(5,6) | right SPG | left SPG | 0.066 | 0.75 |  |
| B(6,6) | right SPG | right SPG | 0.083 | 0.67 |  |
| B(8,6) | right SPG | right vlPFC | -0.065 | 0.72 |  |
| B(1,7) | left vlPFC | left dlPFC | -0.105 | 0.86 |  |
| B(3,7) | left vlPFC | left IPG | -0.06 | 0.75 |  |
| B(5,7) | left vlPFC | left SPG | -0.116 | 0.91 |  |
| B(7,7) | left vlPFC | left vlPFC | -0.166 | 0.85 |  |
| B(8,7) | left vlPFC | right vlPFC | 0.12 | 0.88 |  |
| B(2,8) | right vlPFC | right dlPFC | -0.03 | 0.63 |  |
| B(4,8) | right vlPFC | right IPG | -0.023 | 0.61 |  |
| B(6,8) | right vlPFC | right SPG | -0.016 | 0.57 |  |
| B(7,8) | right vlPFC | left vlPFC | -0.036 | 0.65 |  |
| B(8,8) | right vlPFC | right vlPFC | -0.008 | 0.52 |  |
