# Supplementary material for: Disrupted Frontoparietal Dynamics in Neurofibromatosis Type 1: Reduced Sensitivity and Atypical Modulation During Working Memory
Source: Hum Brain Mapp. 2026 Feb 3;47(2):e70464. doi: 10.1002/hbm.70464 (PMC12865864; doi:10.1002/hbm.70464)
Supplement: Supplementary file 3 — Supporting Information: 3. hbm70464‐sup‐0003‐Supinfo3.docx [file HBM-47-e70464-s002.docx]

**Supplementary material 3**

Precise parameter values and their posterior probabilities for endogenous and modulatory connectivity, reported in the main manuscript. VOIs were positioned at peak group differences (controls>NF1 mass univariate contrast).

| Supplementary Table 3.1 The precise values of all connections from the A-matrix, reported in the main manuscript. VOIs were positioned at peak group differences (controls>NF1 mass univariate contrast). Ep = expected value of the parameter. Ppost = posterior probability of the parameter. | | | | | |
| --- | --- | --- | --- | --- | --- |
| **Parameter** | **Origin** | **Destination** | **Ep** | **Ppost** | **Ppost > 95%** |
| **Covariate 1: Shared connectivity** | | | | | |
| A(1,1) | left dlPFC | left dlPFC | -0.531 | 1 | * |
| A(2,1) | left dlPFC | right dlPFC | 0.128 | 1 | * |
| A(3,1) | left dlPFC | left IPG | 0 | 0 |  |
| A(5,1) | left dlPFC | left SPG | 0 | 0 |  |
| A(7,1) | left dlPFC | left vlPFC | 0.058 | 1 | * |
| A(1,2) | right dlPFC | left dlPFC | -0.028 | 0.59 |  |
| A(2,2) | right dlPFC | right dlPFC | -0.28 | 1 | * |
| A(4,2) | right dlPFC | right IPG | 0 | 0 |  |
| A(6,2) | right dlPFC | right SPG | 0 | 0 |  |
| A(8,2) | right dlPFC | right vlPFC | 0 | 0 |  |
| A(1,3) | left IPG | left dlPFC | 0.129 | 1 | * |
| A(3,3) | left IPG | left IPG | -0.276 | 1 | * |
| A(4,3) | left IPG | right IPG | 0 | 0 |  |
| A(5,3) | left IPG | left SPG | 0.162 | 1 | * |
| A(7,3) | left IPG | left vlPFC | 0.055 | 1 | * |
| A(2,4) | right IPG | right dlPFC | 0.199 | 1 | * |
| A(3,4) | right IPG | left IPG | 0.185 | 1 | * |
| A(4,4) | right IPG | right IPG | -0.348 | 1 | * |
| A(6,4) | right IPG | right SPG | 0.191 | 1 | * |
| A(8,4) | right IPG | right vlPFC | 0 | 0 |  |
| A(1,5) | left SPG | left dlPFC | 0 | 0 |  |
| A(3,5) | left SPG | left IPG | 0 | 0 |  |
| A(5,5) | left SPG | left SPG | -0.293 | 1 | * |
| A(6,5) | left SPG | right SPG | 0 | 0 |  |
| A(7,5) | left SPG | left vlPFC | -0.065 | 1 | * |
| A(2,6) | right SPG | right dlPFC | 0 | 0 |  |
| A(4,6) | right SPG | right IPG | 0 | 0 |  |
| A(5,6) | right SPG | left SPG | 0.075 | 1 | * |
| A(6,6) | right SPG | right SPG | -0.273 | 1 | * |
| A(8,6) | right SPG | right vlPFC | 0.074 | 1 | * |
| A(1,7) | left vlPFC | left dlPFC | 0.226 | 1 | * |
| A(3,7) | left vlPFC | left IPG | 0.091 | 1 | * |
| A(5,7) | left vlPFC | left SPG | 0 | 0 |  |
| A(7,7) | left vlPFC | left vlPFC | -0.466 | 1 | * |
| A(8,7) | left vlPFC | right vlPFC | 0.199 | 1 | * |
| A(2,8) | right vlPFC | right dlPFC | 0.132 | 1 | * |
| A(4,8) | right vlPFC | right IPG | 0 | 0 |  |
| A(6,8) | right vlPFC | right SPG | 0.078 | 1 | * |
| A(7,8) | right vlPFC | left vlPFC | 0.128 | 1 | * |
| A(8,8) | right vlPFC | right vlPFC | -0.324 | 1 | * |
| **Covariate 2: Effect of NF1 diagnosis** | | | | | |
| A(1,1) | left dlPFC | left dlPFC | 0.108 | 1 | * |
| A(2,1) | left dlPFC | right dlPFC | 0.094 | 1 | * |
| A(3,1) | left dlPFC | left IPG | 0 | 0 |  |
| A(5,1) | left dlPFC | left SPG | 0 | 0 |  |
| A(7,1) | left dlPFC | left vlPFC | 0.054 | 1 | * |
| A(1,2) | right dlPFC | left dlPFC | 0 | 0 |  |
| A(2,2) | right dlPFC | right dlPFC | 0 | 0 |  |
| A(4,2) | right dlPFC | right IPG | 0 | 0 |  |
| A(6,2) | right dlPFC | right SPG | 0.057 | 1 | * |
| A(8,2) | right dlPFC | right vlPFC | 0 | 0 |  |
| A(1,3) | left IPG | left dlPFC | -0.11 | 1 | * |
| A(3,3) | left IPG | left IPG | 0.087 | 1 | * |
| A(4,3) | left IPG | right IPG | -0.082 | 1 | * |
| A(5,3) | left IPG | left SPG | -0.115 | 1 | * |
| A(7,3) | left IPG | left vlPFC | -0.058 | 1 | * |
| A(2,4) | right IPG | right dlPFC | 0 | 0 |  |
| A(3,4) | right IPG | left IPG | 0 | 0 |  |
| A(4,4) | right IPG | right IPG | 0 | 0 |  |
| A(6,4) | right IPG | right SPG | 0 | 0 |  |
| A(8,4) | right IPG | right vlPFC | -0.062 | 1 | * |
| A(1,5) | left SPG | left dlPFC | 0.048 | 0.78 |  |
| A(3,5) | left SPG | left IPG | 0 | 0 |  |
| A(5,5) | left SPG | left SPG | 0 | 0 |  |
| A(6,5) | left SPG | right SPG | -0.081 | 1 | * |
| A(7,5) | left SPG | left vlPFC | -0.034 | 0.64 |  |
| A(2,6) | right SPG | right dlPFC | 0 | 0 |  |
| A(4,6) | right SPG | right IPG | 0.101 | 1 | * |
| A(5,6) | right SPG | left SPG | 0.082 | 1 | * |
| A(6,6) | right SPG | right SPG | 0 | 0 |  |
| A(8,6) | right SPG | right vlPFC | 0 | 0 |  |
| A(1,7) | left vlPFC | left dlPFC | 0.113 | 1 | * |
| A(3,7) | left vlPFC | left IPG | 0.169 | 1 | * |
| A(5,7) | left vlPFC | left SPG | 0 | 0 |  |
| A(7,7) | left vlPFC | left vlPFC | 0 | 0 |  |
| A(8,7) | left vlPFC | right vlPFC | 0 | 0 |  |
| A(2,8) | right vlPFC | right dlPFC | -0.073 | 1 | * |
| A(4,8) | right vlPFC | right IPG | 0 | 0 |  |
| A(6,8) | right vlPFC | right SPG | 0 | 0 |  |
| A(7,8) | right vlPFC | left vlPFC | 0 | 0 |  |
| A(8,8) | right vlPFC | right vlPFC | 0 | 0 |  |
| **Covariate 3: Age** | | | | | |
| A(1,1) | left dlPFC | left dlPFC | -0.075 | 1 | * |
| A(2,1) | left dlPFC | right dlPFC | 0 | 0 |  |
| A(3,1) | left dlPFC | left IPG | 0 | 0 |  |
| A(5,1) | left dlPFC | left SPG | -0.018 | 1 | * |
| A(7,1) | left dlPFC | left vlPFC | 0 | 0 |  |
| A(1,2) | right dlPFC | left dlPFC | 0.05 | 1 | * |
| A(2,2) | right dlPFC | right dlPFC | 0 | 0 |  |
| A(4,2) | right dlPFC | right IPG | 0 | 0 |  |
| A(6,2) | right dlPFC | right SPG | -0.033 | 1 | * |
| A(8,2) | right dlPFC | right vlPFC | 0 | 0 |  |
| A(1,3) | left IPG | left dlPFC | 0 | 0 |  |
| A(3,3) | left IPG | left IPG | 0 | 0 |  |
| A(4,3) | left IPG | right IPG | -0.012 | 0.65 |  |
| A(5,3) | left IPG | left SPG | 0 | 0 |  |
| A(7,3) | left IPG | left vlPFC | 0 | 0 |  |
| A(2,4) | right IPG | right dlPFC | -0.052 | 1 | * |
| A(3,4) | right IPG | left IPG | 0 | 0 |  |
| A(4,4) | right IPG | right IPG | -0.055 | 1 | * |
| A(6,4) | right IPG | right SPG | 0 | 0 |  |
| A(8,4) | right IPG | right vlPFC | 0 | 0 |  |
| A(1,5) | left SPG | left dlPFC | -0.061 | 1 | * |
| A(3,5) | left SPG | left IPG | 0 | 0 |  |
| A(5,5) | left SPG | left SPG | 0 | 0 |  |
| A(6,5) | left SPG | right SPG | 0 | 0 |  |
| A(7,5) | left SPG | left vlPFC | 0 | 0 |  |
| A(2,6) | right SPG | right dlPFC | 0 | 0 |  |
| A(4,6) | right SPG | right IPG | 0 | 0 |  |
| A(5,6) | right SPG | left SPG | 0 | 0 |  |
| A(6,6) | right SPG | right SPG | 0 | 0 |  |
| A(8,6) | right SPG | right vlPFC | 0 | 0 |  |
| A(1,7) | left vlPFC | left dlPFC | -0.043 | 1 | * |
| A(3,7) | left vlPFC | left IPG | -0.056 | 1 | * |
| A(5,7) | left vlPFC | left SPG | 0.02 | 0.73 |  |
| A(7,7) | left vlPFC | left vlPFC | -0.058 | 1 | * |
| A(8,7) | left vlPFC | right vlPFC | 0 | 0 |  |
| A(2,8) | right vlPFC | right dlPFC | 0 | 0 |  |
| A(4,8) | right vlPFC | right IPG | 0 | 0 |  |
| A(6,8) | right vlPFC | right SPG | -0.047 | 1 | * |
| A(7,8) | right vlPFC | left vlPFC | 0 | 0 |  |
| A(8,8) | right vlPFC | right vlPFC | -0.017 | 0.55 |  |
| **Covariate 4: Sex (1 = male)** | | | | | |
| A(1,1) | left dlPFC | left dlPFC | -0.283 | 1 | * |
| A(2,1) | left dlPFC | right dlPFC | 0 | 0 |  |
| A(3,1) | left dlPFC | left IPG | 0 | 0 |  |
| A(5,1) | left dlPFC | left SPG | 0.158 | 1 | * |
| A(7,1) | left dlPFC | left vlPFC | 0 | 0 |  |
| A(1,2) | right dlPFC | left dlPFC | 0 | 0 |  |
| A(2,2) | right dlPFC | right dlPFC | 0 | 0 |  |
| A(4,2) | right dlPFC | right IPG | 0 | 0 |  |
| A(6,2) | right dlPFC | right SPG | 0.148 | 1 | * |
| A(8,2) | right dlPFC | right vlPFC | 0 | 0 |  |
| A(1,3) | left IPG | left dlPFC | -0.14 | 1 | * |
| A(3,3) | left IPG | left IPG | 0 | 0 |  |
| A(4,3) | left IPG | right IPG | 0 | 0 |  |
| A(5,3) | left IPG | left SPG | -0.141 | 1 | * |
| A(7,3) | left IPG | left vlPFC | 0 | 0 |  |
| A(2,4) | right IPG | right dlPFC | 0 | 0 |  |
| A(3,4) | right IPG | left IPG | -0.099 | 0.73 |  |
| A(4,4) | right IPG | right IPG | 0 | 0 |  |
| A(6,4) | right IPG | right SPG | -0.134 | 1 | * |
| A(8,4) | right IPG | right vlPFC | 0 | 0 |  |
| A(1,5) | left SPG | left dlPFC | 0.134 | 1 | * |
| A(3,5) | left SPG | left IPG | 0.141 | 1 | * |
| A(5,5) | left SPG | left SPG | -0.263 | 1 | * |
| A(6,5) | left SPG | right SPG | 0 | 0 |  |
| A(7,5) | left SPG | left vlPFC | 0 | 0 |  |
| A(2,6) | right SPG | right dlPFC | 0 | 0 |  |
| A(4,6) | right SPG | right IPG | 0 | 0 |  |
| A(5,6) | right SPG | left SPG | -0.224 | 1 | * |
| A(6,6) | right SPG | right SPG | -0.092 | 0.59 |  |
| A(8,6) | right SPG | right vlPFC | -0.218 | 1 | * |
| A(1,7) | left vlPFC | left dlPFC | 0 | 0 |  |
| A(3,7) | left vlPFC | left IPG | -0.142 | 1 | * |
| A(5,7) | left vlPFC | left SPG | 0 | 0 |  |
| A(7,7) | left vlPFC | left vlPFC | -0.402 | 1 | * |
| A(8,7) | left vlPFC | right vlPFC | 0.131 | 1 | * |
| A(2,8) | right vlPFC | right dlPFC | 0 | 0 |  |
| A(4,8) | right vlPFC | right IPG | 0 | 0 |  |
| A(6,8) | right vlPFC | right SPG | -0.139 | 1 | * |
| A(7,8) | right vlPFC | left vlPFC | 0 | 0 |  |
| A(8,8) | right vlPFC | right vlPFC | -0.207 | 1 | * |

| Supplementary Table 3.2 The precise values of all connections from the A-matrix, reported in the main manuscript. VOIs were positioned at peak group differences (controls>NF1 mass univariate contrast). Ep = expected value of the parameter. Ppost = posterior probability of the parameter. | | | | | |
| --- | --- | --- | --- | --- | --- |
| **Parameter** | **Origin** | **Destination** | **Ep** | **Pp** | **Pp > 95%** |
| **Covariate 1: Shared connectivity** | | | | | |
| B(1,1) | left dlPFC | left dlPFC | -0.01 | 0.55 |  |
| B(2,1) | left dlPFC | right dlPFC | -0.111 | 0.99 | * |
| B(3,1) | left dlPFC | left IPG | 0.055 | 0.86 |  |
| B(5,1) | left dlPFC | left SPG | -0.044 | 0.83 |  |
| B(7,1) | left dlPFC | left vlPFC | -0.004 | 0.54 |  |
| B(1,2) | right dlPFC | left dlPFC | 0.037 | 0.75 |  |
| B(2,2) | right dlPFC | right dlPFC | 0.029 | 0.64 |  |
| B(4,2) | right dlPFC | right IPG | -0.035 | 0.77 |  |
| B(6,2) | right dlPFC | right SPG | 0.016 | 0.63 |  |
| B(8,2) | right dlPFC | right vlPFC | -0.014 | 0.62 |  |
| B(1,3) | left IPG | left dlPFC | -0.08 | 0.95 |  |
| B(3,3) | left IPG | left IPG | 0.037 | 0.7 |  |
| B(4,3) | left IPG | right IPG | 0.014 | 0.64 |  |
| B(5,3) | left IPG | left SPG | -0.075 | 0.95 |  |
| B(7,3) | left IPG | left vlPFC | -0.059 | 0.9 |  |
| B(2,4) | right IPG | right dlPFC | -0.196 | 1 | * |
| B(3,4) | right IPG | left IPG | -0.063 | 0.87 |  |
| B(4,4) | right IPG | right IPG | 0.049 | 0.7 |  |
| B(6,4) | right IPG | right SPG | -0.08 | 0.93 |  |
| B(8,4) | right IPG | right vlPFC | 0.026 | 0.69 |  |
| B(1,5) | left SPG | left dlPFC | 0.001 | 0.51 |  |
| B(3,5) | left SPG | left IPG | 0.065 | 0.87 |  |
| B(5,5) | left SPG | left SPG | 0.183 | 0.97 | * |
| B(6,5) | left SPG | right SPG | -0.056 | 0.85 |  |
| B(7,5) | left SPG | left vlPFC | 0.031 | 0.71 |  |
| B(2,6) | right SPG | right dlPFC | 0.141 | 0.98 | * |
| B(4,6) | right SPG | right IPG | -0.038 | 0.73 |  |
| B(5,6) | right SPG | left SPG | 0.018 | 0.62 |  |
| B(6,6) | right SPG | right SPG | 0.063 | 0.74 |  |
| B(8,6) | right SPG | right vlPFC | 0.033 | 0.72 |  |
| B(1,7) | left vlPFC | left dlPFC | 0.035 | 0.71 |  |
| B(3,7) | left vlPFC | left IPG | -0.108 | 0.97 | * |
| B(5,7) | left vlPFC | left SPG | 0.102 | 0.95 | * |
| B(7,7) | left vlPFC | left vlPFC | -0.072 | 0.76 |  |
| B(8,7) | left vlPFC | right vlPFC | 0.017 | 0.63 |  |
| B(2,8) | right vlPFC | right dlPFC | 0.038 | 0.71 |  |
| B(4,8) | right vlPFC | right IPG | 0.023 | 0.65 |  |
| B(6,8) | right vlPFC | right SPG | 0.071 | 0.88 |  |
| B(7,8) | right vlPFC | left vlPFC | 0.068 | 0.87 |  |
| B(8,8) | right vlPFC | right vlPFC | -0.05 | 0.68 |  |
| **Covariate 2: Effect of NF1 diagnosis** | | | | | |
| B(1,1) | left dlPFC | left dlPFC | -0.091 | 0.86 |  |
| B(2,1) | left dlPFC | right dlPFC | 0.019 | 0.65 |  |
| B(3,1) | left dlPFC | left IPG | 0.049 | 0.84 |  |
| B(5,1) | left dlPFC | left SPG | 0.017 | 0.65 |  |
| B(7,1) | left dlPFC | left vlPFC | 0.058 | 0.9 |  |
| B(1,2) | right dlPFC | left dlPFC | 0.052 | 0.83 |  |
| B(2,2) | right dlPFC | right dlPFC | 0.047 | 0.74 |  |
| B(4,2) | right dlPFC | right IPG | -0.003 | 0.52 |  |
| B(6,2) | right dlPFC | right SPG | 0.02 | 0.67 |  |
| B(8,2) | right dlPFC | right vlPFC | 0.069 | 0.94 |  |
| B(1,3) | left IPG | left dlPFC | -0.068 | 0.91 |  |
| B(3,3) | left IPG | left IPG | 0.007 | 0.55 |  |
| B(4,3) | left IPG | right IPG | 0.021 | 0.69 |  |
| B(5,3) | left IPG | left SPG | 0.06 | 0.91 |  |
| B(7,3) | left IPG | left vlPFC | -0.031 | 0.76 |  |
| B(2,4) | right IPG | right dlPFC | 0.006 | 0.54 |  |
| B(3,4) | right IPG | left IPG | -0.173 | 1 | * |
| B(4,4) | right IPG | right IPG | -0.12 | 0.9 |  |
| B(6,4) | right IPG | right SPG | -0.05 | 0.83 |  |
| B(8,4) | right IPG | right vlPFC | -0.15 | 1 | * |
| B(1,5) | left SPG | left dlPFC | 0.09 | 0.92 |  |
| B(3,5) | left SPG | left IPG | 0.056 | 0.84 |  |
| B(5,5) | left SPG | left SPG | -0.075 | 0.79 |  |
| B(6,5) | left SPG | right SPG | 0.015 | 0.62 |  |
| B(7,5) | left SPG | left vlPFC | 0.007 | 0.56 |  |
| B(2,6) | right SPG | right dlPFC | 0.027 | 0.67 |  |
| B(4,6) | right SPG | right IPG | -0.037 | 0.74 |  |
| B(5,6) | right SPG | left SPG | 0.041 | 0.75 |  |
| B(6,6) | right SPG | right SPG | 0 | 0.5 |  |
| B(8,6) | right SPG | right vlPFC | 0.053 | 0.84 |  |
| B(1,7) | left vlPFC | left dlPFC | 0.041 | 0.76 |  |
| B(3,7) | left vlPFC | left IPG | 0.023 | 0.66 |  |
| B(5,7) | left vlPFC | left SPG | -0.061 | 0.85 |  |
| B(7,7) | left vlPFC | left vlPFC | 0.107 | 0.87 |  |
| B(8,7) | left vlPFC | right vlPFC | -0.088 | 0.96 | * |
| B(2,8) | right vlPFC | right dlPFC | -0.008 | 0.55 |  |
| B(4,8) | right vlPFC | right IPG | -0.056 | 0.85 |  |
| B(6,8) | right vlPFC | right SPG | -0.033 | 0.73 |  |
| B(7,8) | right vlPFC | left vlPFC | 0.051 | 0.83 |  |
| B(8,8) | right vlPFC | right vlPFC | -0.023 | 0.59 |  |
| **Covariate 3: Age** | | | | | |
| B(1,1) | left dlPFC | left dlPFC | 0.02 | 0.69 |  |
| B(2,1) | left dlPFC | right dlPFC | 0.027 | 0.9 |  |
| B(3,1) | left dlPFC | left IPG | -0.01 | 0.69 |  |
| B(5,1) | left dlPFC | left SPG | 0.027 | 0.92 |  |
| B(7,1) | left dlPFC | left vlPFC | -0.008 | 0.66 |  |
| B(1,2) | right dlPFC | left dlPFC | 0.014 | 0.74 |  |
| B(2,2) | right dlPFC | right dlPFC | 0.017 | 0.69 |  |
| B(4,2) | right dlPFC | right IPG | -0.004 | 0.58 |  |
| B(6,2) | right dlPFC | right SPG | 0.019 | 0.83 |  |
| B(8,2) | right dlPFC | right vlPFC | 0.013 | 0.75 |  |
| B(1,3) | left IPG | left dlPFC | 0.076 | 1 | * |
| B(3,3) | left IPG | left IPG | -0.029 | 0.81 |  |
| B(4,3) | left IPG | right IPG | -0.007 | 0.65 |  |
| B(5,3) | left IPG | left SPG | -0.02 | 0.84 |  |
| B(7,3) | left IPG | left vlPFC | 0.042 | 0.98 | * |
| B(2,4) | right IPG | right dlPFC | 0.001 | 0.52 |  |
| B(3,4) | right IPG | left IPG | 0.019 | 0.75 |  |
| B(4,4) | right IPG | right IPG | -0.01 | 0.6 |  |
| B(6,4) | right IPG | right SPG | -0.021 | 0.8 |  |
| B(8,4) | right IPG | right vlPFC | 0.027 | 0.88 |  |
| B(1,5) | left SPG | left dlPFC | 0.029 | 0.86 |  |
| B(3,5) | left SPG | left IPG | -0.034 | 0.92 |  |
| B(5,5) | left SPG | left SPG | 0.046 | 0.9 |  |
| B(6,5) | left SPG | right SPG | -0.006 | 0.61 |  |
| B(7,5) | left SPG | left vlPFC | -0.025 | 0.87 |  |
| B(2,6) | right SPG | right dlPFC | 0.002 | 0.53 |  |
| B(4,6) | right SPG | right IPG | -0.006 | 0.6 |  |
| B(5,6) | right SPG | left SPG | -0.019 | 0.79 |  |
| B(6,6) | right SPG | right SPG | 0.028 | 0.77 |  |
| B(8,6) | right SPG | right vlPFC | 0.012 | 0.7 |  |
| B(1,7) | left vlPFC | left dlPFC | -0.062 | 0.99 | * |
| B(3,7) | left vlPFC | left IPG | -0.013 | 0.69 |  |
| B(5,7) | left vlPFC | left SPG | 0.007 | 0.6 |  |
| B(7,7) | left vlPFC | left vlPFC | 0.008 | 0.57 |  |
| B(8,7) | left vlPFC | right vlPFC | -0.024 | 0.88 |  |
| B(2,8) | right vlPFC | right dlPFC | 0.005 | 0.57 |  |
| B(4,8) | right vlPFC | right IPG | 0.042 | 0.98 | * |
| B(6,8) | right vlPFC | right SPG | -0.008 | 0.65 |  |
| B(7,8) | right vlPFC | left vlPFC | -0.028 | 0.9 |  |
| B(8,8) | right vlPFC | right vlPFC | -0.002 | 0.52 |  |
| **Covariate 4: Sex (1=male)** | | | | | |
| B(1,1) | left dlPFC | left dlPFC | 0.311 | 0.97 | * |
| B(2,1) | left dlPFC | right dlPFC | 0.103 | 0.84 |  |
| B(3,1) | left dlPFC | left IPG | 0.003 | 0.51 |  |
| B(5,1) | left dlPFC | left SPG | -0.042 | 0.68 |  |
| B(7,1) | left dlPFC | left vlPFC | -0.061 | 0.75 |  |
| B(1,2) | right dlPFC | left dlPFC | 0.229 | 0.99 | * |
| B(2,2) | right dlPFC | right dlPFC | -0.065 | 0.65 |  |
| B(4,2) | right dlPFC | right IPG | 0.104 | 0.87 |  |
| B(6,2) | right dlPFC | right SPG | 0.086 | 0.82 |  |
| B(8,2) | right dlPFC | right vlPFC | -0.081 | 0.81 |  |
| B(1,3) | left IPG | left dlPFC | 0.052 | 0.69 |  |
| B(3,3) | left IPG | left IPG | 0.018 | 0.55 |  |
| B(4,3) | left IPG | right IPG | -0.087 | 0.84 |  |
| B(5,3) | left IPG | left SPG | -0.054 | 0.72 |  |
| B(7,3) | left IPG | left vlPFC | -0.025 | 0.61 |  |
| B(2,4) | right IPG | right dlPFC | 0.039 | 0.63 |  |
| B(3,4) | right IPG | left IPG | 0.129 | 0.86 |  |
| B(4,4) | right IPG | right IPG | -0.299 | 0.95 |  |
| B(6,4) | right IPG | right SPG | -0.087 | 0.77 |  |
| B(8,4) | right IPG | right vlPFC | -0.013 | 0.55 |  |
| B(1,5) | left SPG | left dlPFC | 0.101 | 0.8 |  |
| B(3,5) | left SPG | left IPG | 0.107 | 0.83 |  |
| B(5,5) | left SPG | left SPG | -0.08 | 0.66 |  |
| B(6,5) | left SPG | right SPG | -0.01 | 0.54 |  |
| B(7,5) | left SPG | left vlPFC | -0.092 | 0.81 |  |
| B(2,6) | right SPG | right dlPFC | 0.014 | 0.54 |  |
| B(4,6) | right SPG | right IPG | 0.036 | 0.62 |  |
| B(5,6) | right SPG | left SPG | 0.128 | 0.86 |  |
| B(6,6) | right SPG | right SPG | 0.177 | 0.81 |  |
| B(8,6) | right SPG | right vlPFC | 0.039 | 0.63 |  |
| B(1,7) | left vlPFC | left dlPFC | 0.023 | 0.58 |  |
| B(3,7) | left vlPFC | left IPG | -0.135 | 0.89 |  |
| B(5,7) | left vlPFC | left SPG | -0.065 | 0.71 |  |
| B(7,7) | left vlPFC | left vlPFC | -0.214 | 0.87 |  |
| B(8,7) | left vlPFC | right vlPFC | 0.088 | 0.81 |  |
| B(2,8) | right vlPFC | right dlPFC | -0.182 | 0.92 |  |
| B(4,8) | right vlPFC | right IPG | 0.03 | 0.61 |  |
| B(6,8) | right vlPFC | right SPG | -0.008 | 0.53 |  |
| B(7,8) | right vlPFC | left vlPFC | 0.236 | 0.98 | * |
| B(8,8) | right vlPFC | right vlPFC | -0.218 | 0.83 |  |
